# pH-Responsive Upconversion Mesoporous Silica Nanospheres for Combined Multimodal Diagnostic Imaging and Targeted Photodynamic and Photothermal Cancer Therapy

**DOI:** 10.1021/acsnano.3c04564

**Published:** 2023-09-13

**Authors:** L. Palanikumar, Mona Kalmouni, Tatiana Houhou, Osama Abdullah, Liaqat Ali, Renu Pasricha, Rainer Straubinger, Sneha Thomas, Ahmed Jawaad Afzal, Francisco N. Barrera, Mazin Magzoub

**Affiliations:** † Biology Program, Division of Science, New York University Abu Dhabi, P.O. Box 129188, Saadiyat Island, Abu Dhabi, United Arab Emirates; ‡ Core Technology Platforms, New York University Abu Dhabi, P.O. Box 129188, Saadiyat Island, Abu Dhabi, United Arab Emirates; § Department of Biochemistry & Cellular and Molecular Biology, University of Tennessee Knoxville, Knoxville, Tennessee 37996, United States

**Keywords:** cancer therapy, diagnostic imaging, mesoporous silica, near-infrared light, photodynamic therapy, photothermal therapy, upconversion

## Abstract

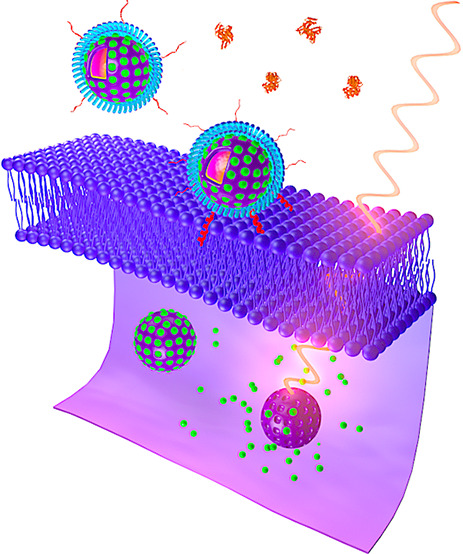

Photodynamic therapy (PDT) and photothermal therapy (PTT) have gained considerable attention as potential alternatives to conventional cancer treatments. However, these approaches remain limited by low solubility, poor stability, and inefficient targeting of many common photosensitizers (PSs) and photothermal agents (PTAs). To overcome the aforementioned limitations, we engineered biocompatible and biodegradable tumor-targeted upconversion nanospheres with imaging capabilities. The multifunctional nanospheres consist of a sodium yttrium fluoride core doped with lanthanides (ytterbium, erbium, and gadolinium) and the PTA bismuth selenide (NaYF_4_:Yb/Er/Gd,Bi_2_Se_3_) enveloped in a mesoporous silica shell that encapsulates a PS, chlorin e6 (Ce6), within its pores. NaYF_4_:Yb/Er converts deeply penetrating near-infrared (NIR) light to visible light, which excites Ce6 to generate cytotoxic reactive oxygen species (ROS), while Bi_2_Se_3_ efficiently converts absorbed NIR light to heat. Additionally, Gd enables magnetic resonance imaging of the nanospheres. The mesoporous silica shell is coated with DPPC/cholesterol/DSPE-PEG to retain the encapsulated Ce6 and prevent serum protein adsorption and macrophage recognition that hinder tumor targeting. Finally, the coat is conjugated to the acidity-triggered rational membrane (ATRAM) peptide, which promotes specific and efficient internalization into malignant cells in the mildly acidic microenvironment of tumors. The nanospheres facilitated tumor magnetic resonance and thermal and fluorescence imaging and exhibited potent NIR laser light-induced anticancer effects *in vitro* and *in vivo* via combined ROS production and localized hyperthermia, with negligible toxicity to healthy tissue, hence markedly extending survival. Our results demonstrate that the ATRAM-functionalized, lipid/PEG-coated upconversion mesoporous silica nanospheres (ALUMSNs) offer multimodal diagnostic imaging and targeted combinatorial cancer therapy.

Traditional cancer treatments, such as chemotherapy, radiotherapy, and surgery, suffer from a number of issues that severely limit their clinical efficacy, including a range of side-effects and complications, immunosuppression, development of multidrug resistance (MDR) phenotypes, recurrence, and metastasis.^1−3^ As a result, new therapeutic approaches are urgently needed to supplement or replace existing cancer treatments. Foremost among these alternatives are noninvasive light-based therapies, photodynamic therapy (PDT) and photothermal therapy (PTT), which have gained considerable attention as potentially safe and effective modalities.^4,5^ PDT uses laser irradiation to activate a photosensitizer (PS) that subsequently generates cytotoxic reactive oxygen species (ROS), through a series of photochemical reactions, to induce apoptosis in cancer cells, while in PTT, a photothermal agent (PTA) converts absorbed light into heat, and the resulting hyperthermia leads to the partial or complete ablation of tumor tissue.^5,6^

Despite their enormous therapeutic potential, PDT and PTT currently have some drawbacks. Many of the common PS and PTA molecules are characterized by poor solubility, rapid *in vivo* degradation and clearance, and lack of tumor specificity.^5,7^ These characteristics are problematic given that ROS are highly reactive and consequently have a very short lifetime (<40 ns) and limited radius of action (∼20 nm) in cellular milieu, which necessitates localization of sufficient amounts of PS molecules to tumor tissue for effective PDT.^7,8^ Likewise, the localized hyperthermia required for PTT is dependent on significant accumulation of PTAs within tumors.^6,7^ Beyond the issues with PSs and PTAs, certain properties of tumors and cancer cells serve to attenuate the potency of PDT and PTT. For instance, since PSs use molecular oxygen to generate ROS, the hypoxic microenvironment of solid tumors can greatly impair PDT.^9−11^ Another challenge is that hyperthermia often leads to overexpression of heat shock proteins, as part of the stress response, which confers a degree of thermotolerance to cancer cells that diminishes the effects of PTT.^12−14^

The current limitations of PDT and PTT have impelled the development of nanocarriers for more efficient tumor delivery of PS and PTA molecules.^5,7,15^ A particularly promising strategy is nanocarrier-mediated simultaneous delivery of PSs and PTAs as means of combining the two forms of phototherapy in order to synergistically enhance their antitumor effects.^5,15^ The advantage of this approach is that PTT-induced hyperthermia can facilitate accumulation of PS molecules and molecular oxygen in tumor tissue by boosting local blood flow, which serves to improve the effectiveness of PDT, while PDT-generated ROS can inactivate heat shock proteins, thereby decreasing the thermotolerance of cancer cells and increasing their susceptibility to PTT.^5,15^ However, currently <1% of intravenously administered NPs accumulate in solid tumors.^16,17^ This is due, in large part, to serum protein adsorption to the surface of nanocarriers while in circulation.^18,19^ Besides destabilizing nanocarriers, adsorbed serum proteins trigger an immune response that leads to rapid blood clearance, all of which hinders accumulation in tumors.^20,21^ Finally, for the small fraction of nanocarriers that does reach the target tumor tissue, uptake into cancer cells represents a major challenge. The primary cellular internalization route for the majority of nanocarriers is endocytosis, but escape from endosomes is highly inefficient, with the bulk of endocytosed nanocarriers becoming entrapped in degradative acidic endocytic compartments or undergoing exocytosis.^17,22^

Here, we have developed multifunctional nanospheres that resolve the issues associated with PDT and PTT. These biocompatible and biodegradable core–shell nanospheres consist of a lanthanide- and PTA-doped upconversion core within a PS-loaded mesoporous silica shell. The shell is wrapped with a lipid/PEG bilayer that is conjugated to the tumor-targeting acidity-triggered rational membrane (ATRAM) peptide.^23^ The ATRAM-functionalized, lipid/PEG-coated upconversion mesoporous silica nanospheres (ALUMSNs) enable tumor detection and monitoring via magnetic resonance, thermal, and fluorescence imaging. The ALUMSNs additionally facilitate NIR laser light-induced PDT and PTT to substantially shrink tumors with no detectable systemic toxicity.

## Results and Discussion

### Preparation and Characterization of the Upconversion Mesoporous Silica Nanospheres (UMSNs)

**Figure 1:**
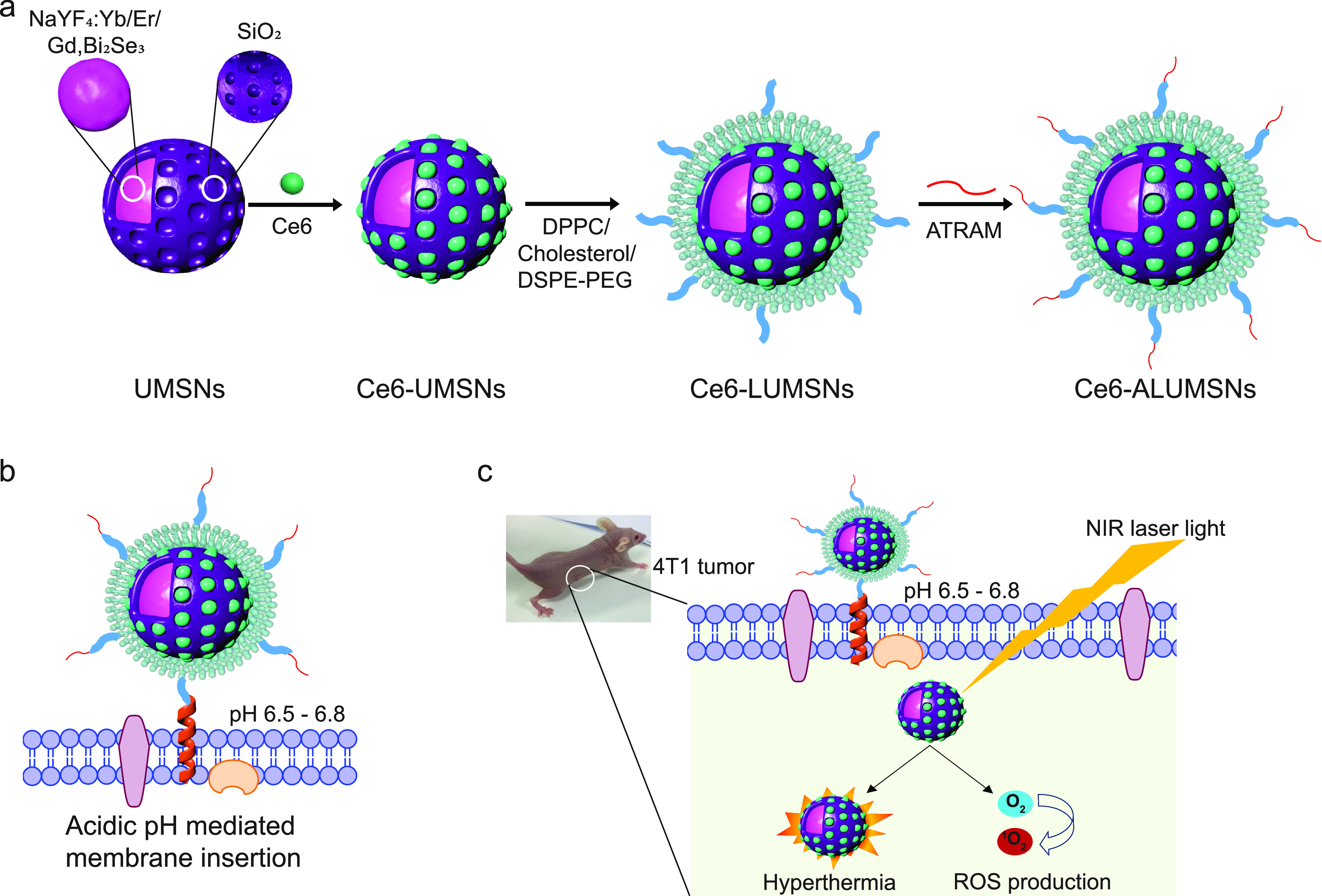
Schematic representation of preparation and mode of action of the tumor-targeted nanospheres. (a) The nanospheres consist of an upconversion core of sodium yttrium fluoride doped with lanthanides—ytterbium, erbium, and gadolinium—and bismuth selenide (NaYF_4_:Yb/Er/Gd,Bi_2_Se_3_) within a mesoporous silica shell that encapsulates a photosensitizer, chlorin e6 (Ce6), in its pores. The Ce6-loaded upconversion mesoporous silica nanospheres (Ce6-UMSNs) are then “wrapped” with lipid/polyethylene glycol (DPPC/cholesterol/DSPE-PEG_2000_-maleimide). Finally, the Ce6-loaded, lipid/PEG-coated UMSNs (Ce6-LUMSNs) are functionalized with the acidity-triggered rational membrane (ATRAM) peptide. (b) In mildly acidic conditions, ATRAM inserts into lipid bilayers as a transmembrane α-helix. As the membrane insertion p*K*_a_ of ATRAM is 6.5,^23^ the peptide promotes targeting of ATRAM-functionalized Ce6-LUMSNs (Ce6-ALUMSNs) to cancer cells in the mildly acidic (pH ≈ 6.5–6.8) microenvironment of solid tumors.^71^ (c) ALUMSNs are efficiently internalized into tumor cells, where subsequent near-infrared (NIR, 980 nm) laser irradiation of the nanospheres results in substantial cytotoxicity due to the combined effects of hyperthermia and reactive oxygen species (ROS) generation.

**Figure 2:**
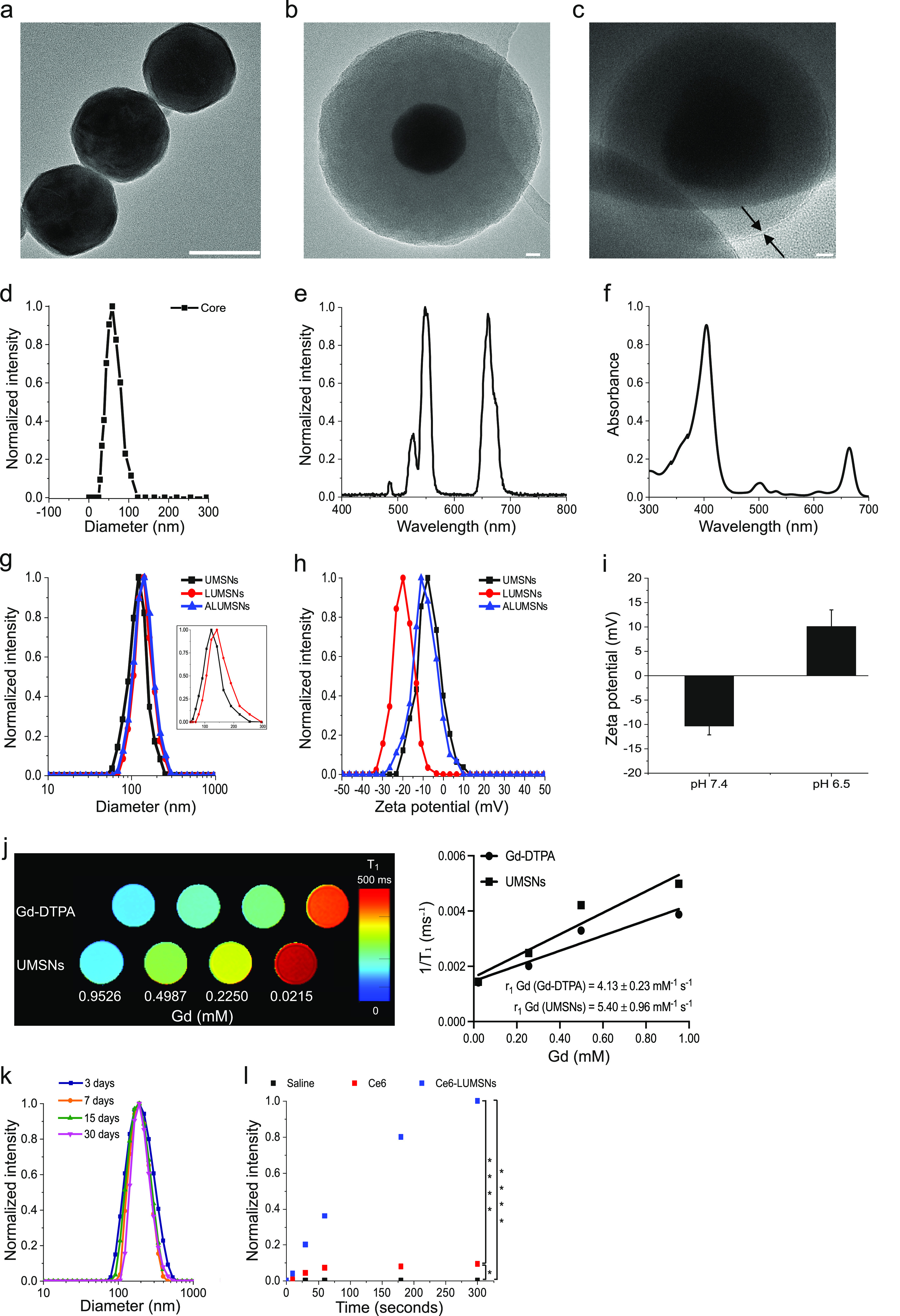
Characterization of the upconversion mesoporous silica nanospheres. (a–c) Transmission electron microscopy (TEM) images of the upconversion core (NaYF_4_:Yb/Er/Gd,Bi_2_Se_3_) (a), upconversion mesoporous silica nanospheres (UMSNs) (b), and lipid/PEG-coated UMSNs (LUMSNs) (c). The arrows in (c) indicate the lipid bilayer. Scale bar in (a) = 50 nm, in (b) and (c) = 10 nm. (d) Size analysis for the upconversion core in 10 mM phosphate buffer (pH 7.4) using dynamic light scattering (DLS). (e) Fluorescence emission spectrum of the upconversion core (λ_ex_ = 980 nm). (f) UV–vis absorption spectrum of chlorin e6 (Ce6) (Soret peak at 404 nm and Q-band at 658 nm).^33^ (g,h) Size distribution analysis (g) and zeta potential measurements (h) for UMSNs, LUMSNs, and ALUMSNs in 10 mM phosphate buffer (pH 7.4). *Inset*: expanded scale to show difference in hydrodynamic diameters of UMSNs and LUMSNs. (i) Comparison of zeta potentials of ATRAM-functionalized LUMSNs (ALUMSNs) at pH 7.4 and 6.5. (j) *T*_1_ maps (*left*) and the relaxation rates (1/*T*_1_) (*right*) of UMSNs compared to commercial Gd-DTPA (at the same concentrations of the lanthanide) (*n* = 3). (k) Colloidal stability analysis for LUMSNs in complete cell culture medium (RPMI 1640 containing 10% FBS, pH 7.4) over 30 days at 37 °C. (l) Comparison of ROS production capability of Ce6-LUMSNs and free Ce6, at the same Ce6 concentration (0.5 μg/mL) and NIR laser irradiation power density and duration (1.0 W/cm^2^, 5 min), monitored in 10 mM phosphate buffer (pH 7.4) using the fluorescent probe Singlet Oxygen Sensor Green (SOSG).^58^ *P < 0.05, ****P < 0.001 for comparisons with controls or among the different samples.

The core of the upconversion mesoporous silica nanospheres (UMSNs) consists of sodium yttrium fluoride doped with lanthanides (ytterbium, erbium, and gadolinium) and bismuth selenide (NaYF_4_:Yb/Er/Gd,Bi_2_Se_3_) (Figure 1a). Transmission electron microscopy (TEM) and scanning transmission electron microscopy (STEM) images showed a uniform sphere-like upconversion core (Figure 2a; Supporting Figure 1a,b). The composition of the upconversion core was verified using scanning transmission electron microscopy–energy dispersive X-ray spectroscopy (STEM–EDS) mapping (Supporting Figure 1c). The average hydrodynamic diameter of the core was confirmed by dynamic light scattering (DLS) to be ∼60 nm (Figure 2d).

Using a facile synthesis method (Supporting Experimental Section),^24^ the core was enveloped in a mesoporous silica shell (Figure 1a). Mesoporous silica was selected due to its physicochemical properties that make it highly suited for drug delivery applications: excellent biocompatibility and biodegradability, low toxicity, high thermal and chemical stability, large surface area for drug loading by adsorption, tunable pore size to modulate drug release kinetics, and ease of surface modification for increased *in vivo* circulation time and improved targeting.^25−28^ N_2_ adsorption–desorption isotherms showed that the shell has a specific surface area of ∼700 m^2^/g and an average pore diameter of ∼2.3 nm (Supporting Figure 2a), which is within the range reported for other promising mesoporous silica-based drug delivery nanoplatforms.^27,28^ TEM, high-angle annular dark-field scanning transmission electron microscopy (HAAD-STEM), and STEM–EDS confirmed the formation of UMSNs as a core–shell structure, which had a hydrodynamic diameter of 160 ± 10 nm and a zeta potential of −6 mV (Figure 2b,g,h; Supporting Figure 2b–d; Supporting Information Table 1). The photosensitizer (PS) chlorin e6 (Ce6) was encapsulated within the pores of the mesoporous silica shell using a passive entrapment loading technique. By adjusting the feed ratio, a relatively high loading capacity of Ce6 in the UMSNs was achieved (22 wt %; Supporting Information Table 2).

The core NaYF_4_:Yb/Er is excited by near-infrared (NIR) light, which has greater tissue penetration depth, lower autofluorescence, and reduced phototoxicity compared to visible light.^29−32^ Spectroscopic analysis revealed clear overlap between the fluorescence emission of the upconversion core and the absorption of Ce6 at the Q-band at 658 nm (Figure 2e,f).^33^ Therefore, under NIR irradiation, the fluorescence emission from the upconversion core will excite the Ce6 encapsulated within the pores of the UMSNs to generate cytotoxic reactive oxygen species (ROS) (Supporting Figure 3). The photothermal agent (PTA) Bi_2_Se_3_ was additionally incorporated in the core to simultaneously convert absorbed NIR light to heat for thermal imaging and PDT.^34,35^ Finally, by doping the core with Gd, UMSNs can also serve as MRI contrast agents.^36,37^*T*_1_ maps and relaxation rate (1/*T*_1_) plots^38,39^ demonstrate that UMSNs consistently yielded greater contrast enhancement compared to the clinically used contrast agent Gd-DTPA (at the same concentrations of the lanthanide; Figure 2j).

### Characterization of Lipid/PEG-Coated UMSNs (LUMSNs)

Nanocarriers for drug delivery applications are typically coated with lipid bilayers to improve biocompatibility, colloidal stability, and controlled therapeutic cargo release.^40,41^ Lipid coatings also offer the advantage that they can be readily functionalized to achieve tissue- and cell-specific targeting.^42^ Moreover, the lipid bilayer coat can be doped with an inert, water-soluble polymer, such as polyethylene glycol (PEG), that reduces aggregation and minimizes interactions with serum components that mediate the phagocytic clearance.^43^

We used established protocols to coat the surface of Ce6-free and Ce6-loaded UMSNs with a bilayer consisting of DPPC, cholesterol, and DSPE-PEG_2000_-maleimide (Figure 1a; Supporting Experimental Section).^40^ Contacts between the bilayer coat and UMSN are likely stabilized by van der Waals and electrostatic interactions between the phospholipid headgroups and the negatively charged UMSN.^44^ The phospholipid DPPC was chosen due to its saturated acyl chains, as unsaturated lipids have been shown to reduce the long-term colloidal stability of lipid-coated mesoporous silica nanocarriers.^45,46^ Cholesterol was used to decrease the bilayer fluidity and, in turn, reduce the baseline leakage of the Ce6 encapsulated in the pores of the UMSNs.^41,47^ Finally, PEGylated DSPE was added to increase the *in vivo* circulation half-life of the nanospheres,^46,48^ with the maleimide group on the PEG facilitating functionalization with a cancer-targeting moiety. The composition of the bilayer (DPPC/cholesterol/DSPE-PEG_2000_-maleimide at a 77.5:20:2.5 molar ratio) was chosen, as it was reported to yield high colloidal stability and cargo loading as well as negligible baseline cargo leakage.^40,46^

Transmission electron microscopy (TEM) images showed that the lipid/PEG layered over the surface of UMSNs (Figure 2c). Coating was further confirmed by DLS measurements, which showed a homogeneous colloidal solution (polydispersity index = 0.11 ± 0.02)^41,45^ of lipid/PEG-coated UMSNs (LUMSNs) that have an expectedly larger hydrodynamic diameter (180 ± 10 nm) compared to UMSNs (Figure 2g; Supporting Information Table 1). This translates to a lipid/PEG bilayer coat thickness of ∼10 nm. It should be noted that the discrepancy in the lipid/PEG bilayer thickness between the TEM images (Figure 2c) and DLS measurements (Figure 2g) is likely due to unavoidable differences in the experimental conditions (aqueous solution vs dehydrated sample for DLS vs TEM, respectively) and the fact that PEG is not visible by electron microscopy.^40,49^ Additionally, the zeta potential changed from −6 to −20 mV after lipid/PLGA coating (Figure 2h; Supporting Information Table 1), which is in agreement with the values reported for other lipid-coated mesoporous silica nanocarriers.^40^

The colloidal stability of LUMSNs was assessed to gauge their suitability for tumor targeting and cancer therapy applications.^50,51^ There was no change in the hydrodynamic diameter of the nanospheres in 10 mM phosphate buffer at pH 7.4 (180 ± 10 nm), 50 mM sodium acetate buffer solution at pH 5.5 (180 ± 15 nm), or complete cell culture medium (RPMI 1640, 10% FBS, pH 7.4) (183 ± 10 nm) over 72 h (Supporting Figure 4; Supporting Information Table 1). Importantly, long-term monitoring of LUMSNs revealed that they are stable for at least 1 month in complete medium (Figure 2k). These results indicate that coating UMSNs with lipid/PEG leads to colloidal stabilization and inhibits adsorption of serum proteins and suggests the nanospheres are able to maintain an appropriate size during *in vivo* circulation, which would aid in tumor localization and internalization into cancer cells.^52,53^

Formation of a serum protein corona during circulation not only destabilizes nanocarriers but also triggers an immune response and leads to rapid elimination, preventing accumulation in target tumor tissue.^18,21^ Therefore, we further analyzed serum protein adsorption to the surface of the nanospheres using quantitative proteomics (Supporting Figure 5; Supporting Information Table 3). Following 72 h incubation of UMSNs and LUMSNs in complete cell culture medium, we isolated the adsorbed serum proteins and quantified them using reversed-phase liquid chromatography–tandem mass spectrometry (RPLC-MS/MS) with label-free quantification (LFQ).^54^ The analysis showed noticeably fewer serum proteins adhered to LUMSNs compared to UMSNs (Supporting Figure 5; Supporting Information Table 3). Thus, the lipid/PEG coat effectively blocks formation of a serum protein corona on the LUMSN surface.

### Photodynamic and Photothermal Properties of Ce6-Loaded LUMSNs

Ce6 is a widely used, FDA-approved, second-generation PS that is characterized by high singlet oxygen (^1^O_2_) quantum yield and low dark toxicity.^55−57^ However, Ce6 is prone to aggregation in solution, due to the presence of several alkyl groups, which attenuate the PS’s ^1^O_2_ production capacity.^55,56^ Here, to minimize aggregation, we loaded Ce6 into the pores of LUMSNs (see Supporting Experimental Section). We monitored the ROS production capability of Ce6-loaded LUMSNs (Ce6-LUMSNs) using the fluorescent probe Singlet Oxygen Sensor Green (SOSG).^58^ Following NIR laser illumination, substantially higher ROS were detected in the presence of Ce6-LUMSNs compared to free Ce6, at the same Ce6 concentration and irradiation power density and duration (Figure 2l). This illustrates the capacity of the nanospheres to generate ROS upon exposure to NIR light.

**Figure 3:**
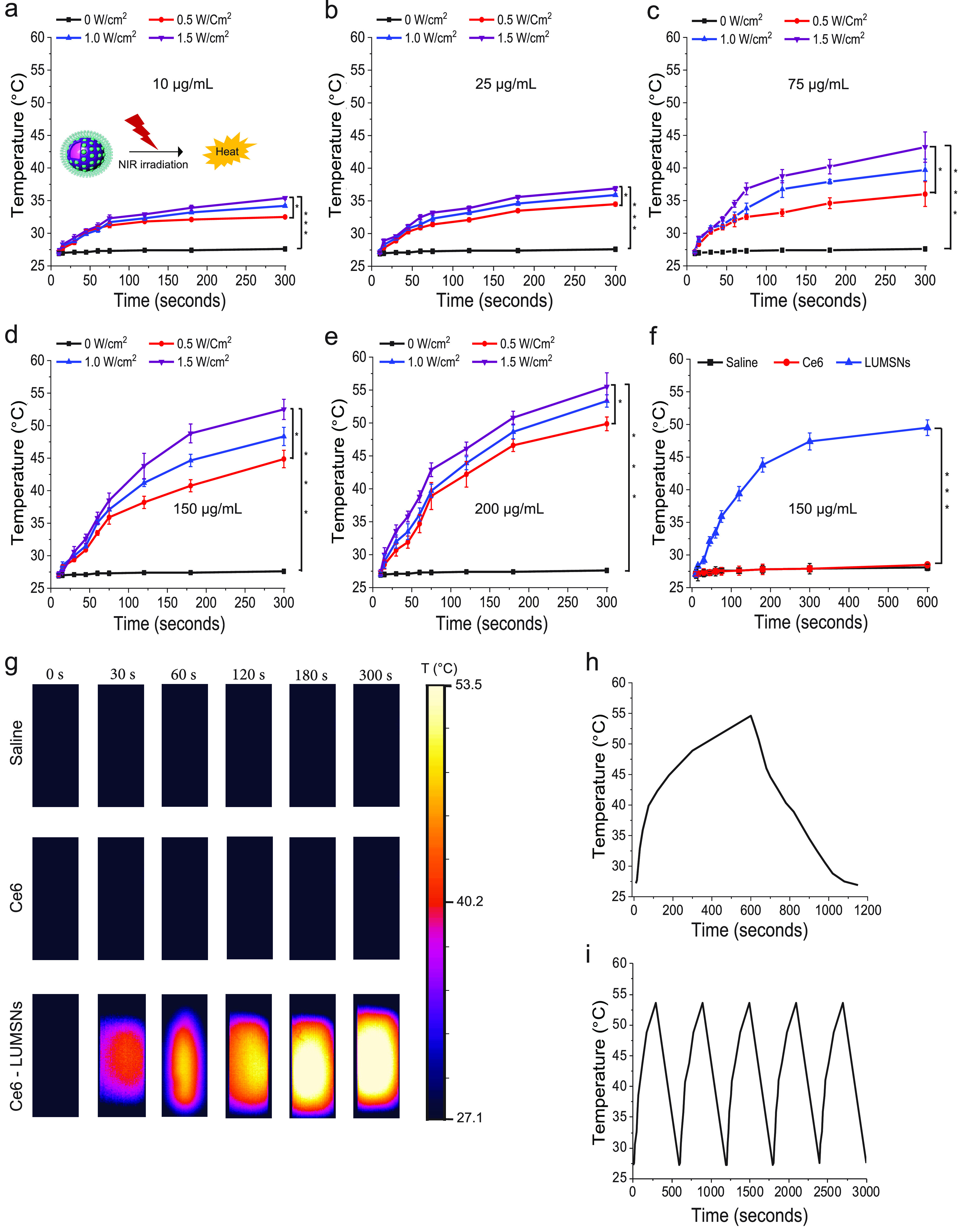
Photothermal properties of Ce6-loaded LUMSNs. (a–e) Temperature increases following NIR laser irradiation (0.5–1.5 W/cm^2^, 5 min) of Ce6-LUMSNs at nanosphere concentrations of 10 (a), 25 (b), 75 (c), 150 (d), and 200 μg/mL (e), in 10 mM phosphate buffer (pH 7.4). (f) Comparison of NIR laser light (1.0 W/cm^2^, 10 min)-induced temperature increases in Ce6 and Ce6-LUMSN samples (33 μg/mL Ce6) in 10 mM phosphate buffer (pH 7.4). (g) Thermal images of saline, Ce6, and Ce6-LUMSN (33 μg/mL Ce6) samples illuminated with NIR laser light (1.5 W/cm^2^) for 5 min. (h) Photothermal response profile of Ce6-LUMSNs (150 μg/mL nanospheres) subjected to NIR laser irradiation (1.5 W/cm^2^, 10 min) followed by natural cooling. (i) Photothermal stability of Ce6-LUMSNs (150 μg/mL nanospheres) monitored over five consecutive NIR laser irradiation (1.5 W/cm^2^, 5 min) on/off cycles. *P < 0.05, ***P < 0.001 for comparisons with controls or among the different samples.

Biocompatible and nontoxic Bi_2_Se_3_-based nanomaterials are reported to combine strong NIR absorption with high photothermal conversion efficiency.^34,35^ We therefore investigated the temperature changes induced by NIR laser illumination of the PTA-doped LUMSNs in aqueous solution. As expected, no change in temperature was observed in Ce6-LUMSN samples in the absence of NIR light (Figure 3a–e,g). However, upon exposure to 980 nm laser light, the Ce6-LUMSNs showed a robust, nanosphere concentration- and irradiation power density-dependent photothermal response (Figure 3a–e). For instance, at 150 μg/mL LUMSNs with 1.0 W/cm^2^ irradiation for 5 min, the temperature increased from 27.1 ± 0.4 to 48.3 ± 1.4 °C, while at 200 μg/mL LUMSNs with 1.5 W/cm^2^ irradiation for 5 min, the temperature rose to 55.5 ± 2.1 °C (Figure 3d,e). This suggests that the nanospheres can rapidly and efficiently convert NIR laser light into heat of a temperature that is high enough to ablate malignant cells (typically ∼50 °C).^5^ Notably, even with ultralow laser power densities,^59,60^ Ce6-LUMSNs still yielded significant temperature increases (e.g., at a nanosphere concentration of 200 μg/mL, the temperature rose to ∼39–48 °C following exposure to 0.2–0.4 W/cm^2^ irradiation for 5 min; Supporting Figure 6), which is comparable to the responses reported for PTA-based nanomaterials that exhibit high photothermal conversion efficiencies.^34,61,62^ In contrast, a negligible increase in temperature (to ∼28 °C) was recorded in the free Ce6 sample compared to Ce6-LUMSNs under the same experimental conditions (Figure 3f,g), indicating that the photothermal property of LUMSNs is due to the presence of Bi_2_Se_3_. This is supported by the photothermal response profile of the LUMSNs (Figure 3h), which matches that of other Bi_2_Se_3_-based PTT nanomaterials.^35,63^ Additionally, the photothermal stability of LUMSNs was assessed over five laser on/off cycles. The maximum temperature (∼55 °C) was nearly identical over the five successive heating/cooling cycles, underlining the high photostability of the LUMSNs (Figure 3i).^35,61,62^ Together, these results emphasize the PDT and PTT potentials of the designed nanospheres.

### NIR Light-Triggered Cargo Release Profile of LUMSNs

**Figure 4:**
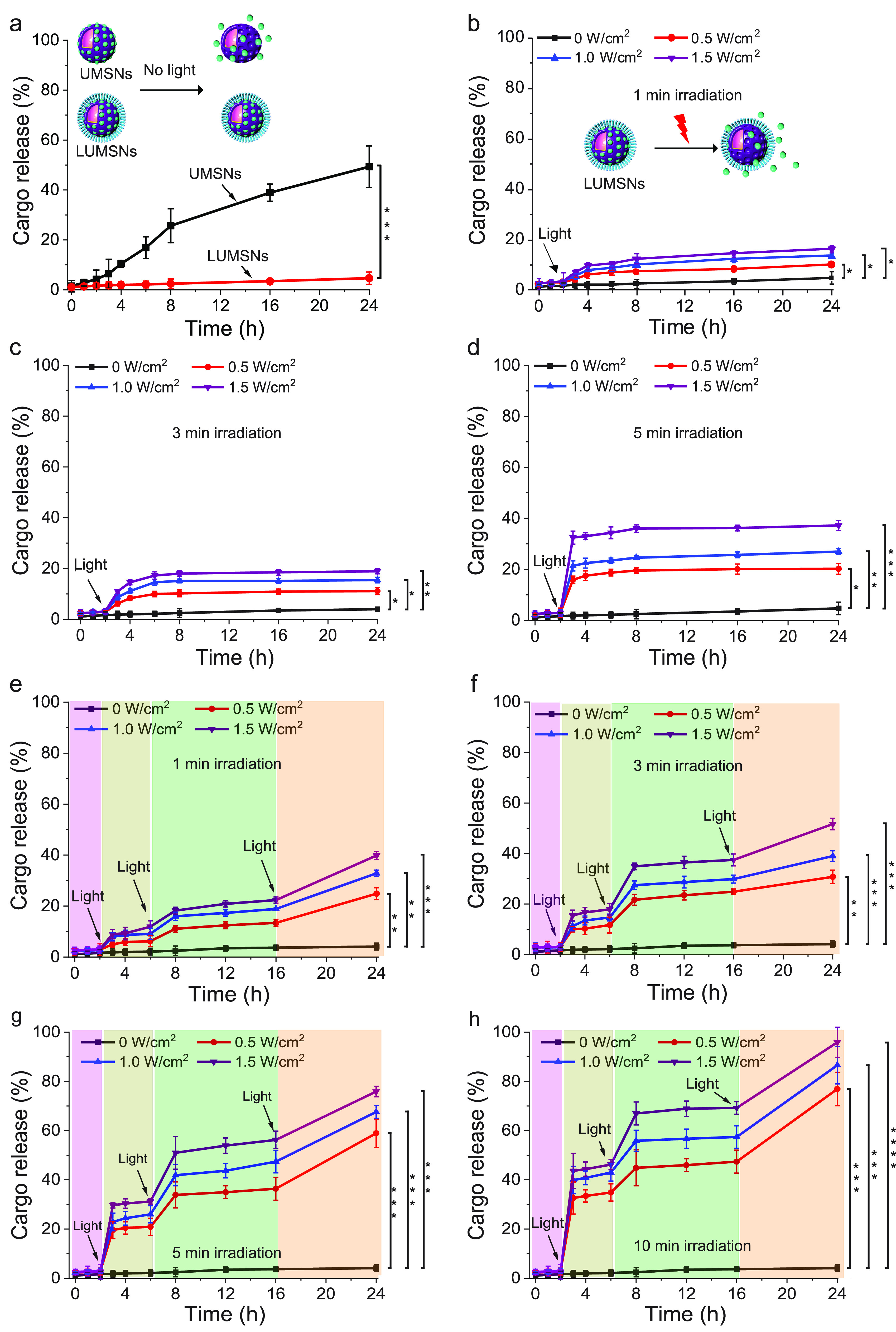
Cargo release profiles of Ce6-loaded LUMSNs in the absence and presence of a stimulus. (a) Release of Ce6 from UMSNs and LUMSNs (50 μg/mL nanospheres) in the absence of NIR laser irradiation. (b–d) NIR laser light-triggered release of Ce6 from LUMSNs (50 μg/mL nanospheres) at varying irradiation power densities (0.5–1.5 W/cm^2^) and durations of 1 (b), 3 (c), or 5 min (d), in 10 mM phosphate buffer (pH 7.4). (e–h) On-demand release of Ce6 from LUMSNs (50 μg/mL nanospheres) due to sequential illumination with NIR laser light at varying irradiation power densities (0.5–1.5 W/cm^2^) and durations of 1 (e), 3 (f), 5 (g), or 10 min (h), in 10 mM phosphate buffer (pH 7.4). *P < 0.05, **P < 0.01, ***P < 0.001, ****P < 0.0001 for comparisons with controls or among the different samples.

In addition to their photodynamic and photothermal properties, we assessed the capacity of the designed nanospheres to function as a controlled release cancer therapeutic delivery platform. In the absence of 980 nm laser irradiation, UMSNs released ∼50% of encapsulated Ce6 over the 24 h duration of measurement due to diffusion of the PS out of the pores of the uncoated nanospheres (Figure 4a). In contrast, stimulus-free leakage of Ce6 cargo from LUMSNs was negligible over the duration of the experiment (Figure 4a). Thus, wrapping the nanospheres with a lipid/PEG coat resulted in a highly stable nanocarrier, which is crucial for preventing premature release and delivering the therapeutic payload to the malignant cells.

Upon exposure to the 980 nm laser with varying irradiation power densities (0.5–1.5 W/cm^2^) and durations (1–5 min), the encapsulated Ce6 was efficiently released from LUMSNs (Figure 4b–d). This effect can be attributed to NIR light-induced hyperthermia increasing the fluidity and permeability of the bilayer coat. The melting temperature (*T*_m_) of the primary phospholipid of the bilayer, DPPC, is ∼41 °C,^64^ while the temperature of the LUMSNs typically rises to >45 °C following irradiation (Figure 3d), which leads to payload release.^65^ Crucially, sequential NIR light illumination (0.5–1.5 W/cm^2^, 1–10 min) of the LUMSNs triggered repeated release of the Ce6 cargo, culminating in a maximum cumulative release of 40–96% (Figure 4e–h). Release of Ce6 within the cancer cells would allow the PS to directly target mitochondria and other intracellular organelles, which are particularly susceptible to the detrimental effects of ROS, leading to a more potent PDT response.^66−68^ Furthermore, the robust NIR light-induced on-demand release of the encapsulated cargo of LUMSNs highlights their potential as a platform for combining phototherapies with chemotherapy.

### Cancer Cell Uptake of ATRAM-Functionalized LUMSNs (ALUMSNs)

For tumor targeting, LUMSNs were functionalized with the pH-responsive acidity-triggered rational membrane (ATRAM) peptide (Figure 1).^69,70^ The interaction of ATRAM (N_t_-CGLAGLAGLLGLEGLLGLPLGLLEGLWLGLELEGN-C_t_) with cellular membranes is highly pH-dependent: ATRAM binds weakly and superficially to membranes, as a largely unstructured peptide, at physiological pH; conversely, the peptide adopts a transmembrane α-helical conformation in lipid bilayers at acidic pH (Figure 1b).^23,69^ ATRAM’s membrane insertion in acidic conditions is driven by the increased hydrophobicity of the peptide due to protonation of its acidic glutamate residues.^23^ Importantly, the peptide’s membrane insertion p*K*_a_ is 6.5,^23^ rendering ATRAM ideally suited for targeting malignant cells in the mildly acidic (pH ≈ 6.5–6.8) microenvironment of solid tumors (Figure 1b, c).^71−73^

We previously established that ATRAM’s membrane insertion occurs via the peptide’s C-terminus.^69^ Thus, LUMSNs were conjugated to ATRAM by covalently coupling the DSPE-PEG-maleimide of the lipid coat to the N-terminal cysteine of the peptide (Supporting Experimental Section). The ATRAM-functionalized LUMSNs (ALUMSNs) were characterized using DLS and zeta potential measurements. As expected, conjugation of the peptide did not appreciably alter the hydrodynamic diameter of the nanospheres significantly (181 ± 10 nm) (Figure 2g; Supporting Information Table 1). However, the zeta potential at pH 7.4 increased from −20 to −11 mV (Figure 2h; Supporting Information Table 1), which confirms conjugation of ATRAM to the LUMSNs. Of relevance, the zeta potential of ALUMSNs falls within the range reported for other highly stable nanocarriers at physiological pH.^66,70,74,75^ Lowering the pH to 6.5 increased the zeta potential of ALUMSNs to +11 mV, without adversely affecting the long-term colloidal stability of the nanospheres (Figure 2i; Supporting Figure 7; Supporting Information Table 1). These results strongly suggest that ALUMSNs would effectively target tumor cells.

**Figure 5:**
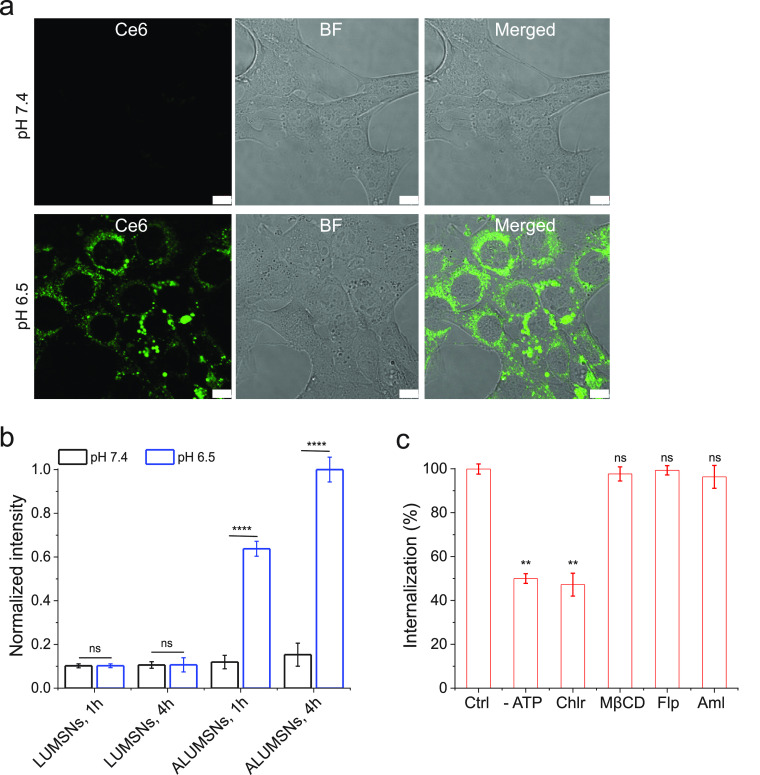
pH-dependent cellular uptake of Ce6-loaded ALUMSNs. (a) Confocal fluorescence microscopy images of 4T1 cells incubated with Ce6-ALUMSNs (0.5 μg/mL Ce6) for 4 h at physiological (*top panels*) or acidic (*lower panels*) pH. Ce6 is pseudocolored green for clarity. Imaging experiments were performed in quadruplicate, and representative images are shown. Scale bar = 10 μm. (b) Flow cytometry quantification of cellular uptake of Ce6-loaded LUMSNs and ALUMSNs (0.5 μg/mL Ce6) in 4T1 cells following treatment for 1 or 4 h at pH 7.4 or 6.5 (*n* = 4). (c) Flow cytometry quantification of cellular uptake of Ce6-ALUMSNs (0.5 μg/mL Ce6) at pH 6.5 in 4T1 cells pretreated with sodium azide and 2-deoxy-d-glucose (-ATP) or with endocytosis inhibitors—chlorpromazine (Chlor), methyl-β-cyclodextrin (MβCD), filipin (Flp), or amiloride (Aml)—compared with uninhibited uptake in control cells (Ctrl) (*n* = 4). **P < 0.01, ****P < 0.0001 or nonsignificant (ns, *P* > 0.05) for comparisons with controls.

The pH-dependent uptake of ALUMSNs in cancer cells was assessed using confocal fluorescence microscopy, TEM, and flow cytometry. Murine breast cancer 4T1 cells were treated with ALUMSNs for 4 h (Figure 5). Confocal microscopy images showed substantially higher cellular internalization and cytosolic localization of ALUMSNs under acidic conditions compared to physiological pH (Figure 5a). Similarly, TEM revealed much greater accumulation of the nanospheres intracellularly following incubation for 4 h at pH 6.5 relative to 7.4 (Supporting Figure 8). The imaging results were confirmed with flow cytometry analysis, which showed ∼6- and ∼9-fold higher uptake at acidic versus physiological pH at 1 and 4 h incubations, respectively (Figure 5b). In contrast, poor uptake of LUMSNs (i.e., in the absence of ATRAM) was observed in 4T1 cells at both pHs (7.4 and 6.5) and incubation times (1 and 4 h; Figure 5b). These results confirm that ATRAM facilitates uptake of ALUMSNs specifically in cells within a mildly acidic environment.

Next, we conducted a series of experiments to elucidate the cellular internalization mechanism(s) of ALUMSNs. Depleting intracellular ATP using sodium azide/deoxyglucose only partially decreased the level of cellular internalization of ALUMSNs (to ∼50% of controls), indicating that the nanospheres are taken up by both energy-dependent (e.g., endocytosis) and energy-independent (i.e., direct translocation) mechanisms (Figure 5c). The direct translocation mechanism likely entails ATRAM-mediated anchoring followed by fusion of the lipid-based coat with the cancer cell membrane and concomitant release of the UMSNs into the cytosol.^70^

In order to determine the nature of the energy-dependent uptake mechanism, the cells were pretreated with specific endocytosis inhibitors: chlorpromazine (clathrin-coated pit formation inhibitor),^76^ methyl-β-cyclodextrin (disrupts lipid-raft-mediated endocytic pathways by depleting plasma membrane cholesterol),^77^ filipin (caveolae-dependent endocytosis inhibitor),^78^ or amiloride (Na^+^/H^+^ exchange inhibitor that blocks micropinocytosis).^79^ Of all the inhibitors tested, only chlorpromazine significantly diminished cellular internalization, which indicates that uptake of ALUMSNs occurs partially via clathrin-mediated endocytosis (Figure 5c). In the case of membrane translocation, ALUMSNs would directly access the cytosol; on the other hand, following uptake by clathrin-mediated endocytosis, acidification of mature endocytic compartments would drive endosome membrane insertion and disruption by ATRAM, similar to other pH-responsive peptides, leading to cytosolic release of ALUMSNs.^80,81^ Thus, the pH-dependent cellular uptake of ALUMSNs occurs by multiple mechanisms, which enable the nanospheres to efficiently enter tumor cells.

### Cancer Cell Toxicity of Ce6-Loaded ALUMSNs

The anticancer efficacy of the designed nanospheres was evaluated using the MTS cell viability assay.^82,83^ In the absence of NIR laser irradiation, treatment with Ce6-free UMSNs or LUMSNs (5–100 μg/mL) did not significantly reduce breast cancer 4T1 cell viability at either physiological or acidic pH (Supporting Figure 9a). Likewise, without NIR laser light, the Ce6-loaded ALUMSNs (Ce6-ALUMSNs) were not toxic to 4T1 cells, up to a Ce6 concentration of 5 μg/mL, at pH 7.4 or 6.5 (Supporting Figure 9b). These results confirm that the nanospheres are biocompatible and therefore suitable for cancer therapy applications.

**Figure 6:**
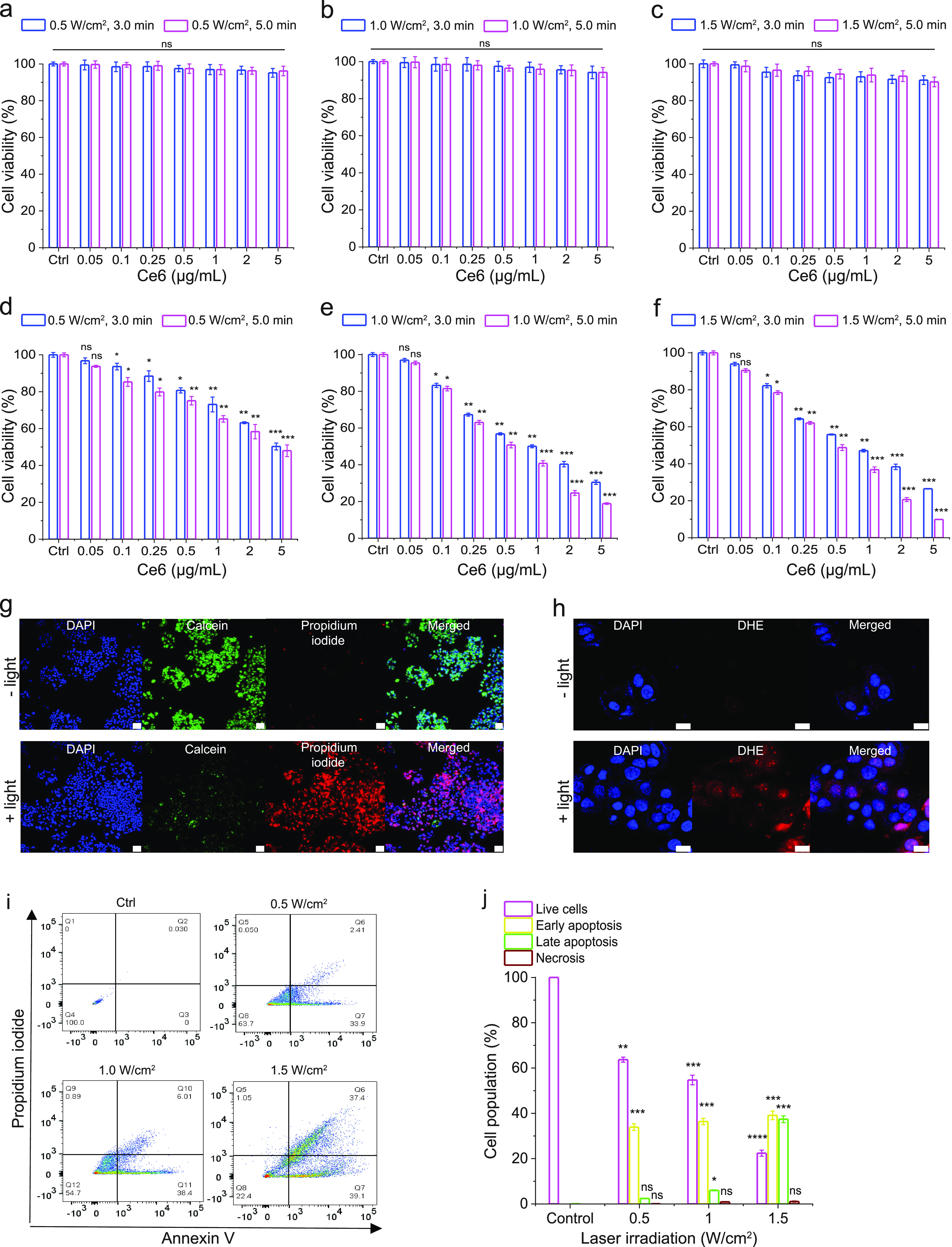
NIR light-triggered cytotoxicity of Ce6-loaded ALUMSNs. (a–f) Cell viability of 4T1 cells treated with Ce6-ALUMSNs (0.05–5 μg/mL Ce6) for 48 h with subsequent exposure to NIR laser light of different irradiation power densities (0.5–1.5 W/cm^2^) and durations (3.0 or 5.0 min) at pH 7.4 (a–c) or 6.5 (d–f). Cell viability in (a–f) was measured using the MTS assay, with the % viability determined form the ratio of the absorbance of the treated cells to the control cells (*n* = 4). (g) Calcein AM/PI staining of 4T1 cells incubated with Ce6-ALUMSNs (0.5 μg/mL Ce6) for 12 h at pH 6.5 in the absence (− light) or presence (+ light) of NIR laser irradiation (1.0 W/cm^2^, 5 min). Scale bar = 50 μm. (h) ROS probe DHE staining of 4T1 cells treated with Ce6-ALUMSNs (0.5 μg/mL Ce6) for 4 h at pH 6.5 in the absence (− light) or presence (+ light) of NIR laser irradiation (1.0 W/cm^2^, 5 min). Scale bar = 10 μm. Confocal laser scanning microscopy imaging experiments in (g,h) were performed in quadruplicate, and representative images are shown. (i) Flow cytometry analysis of annexin V/PI-stained 4T1 cells that were either untreated (control, Ctrl) or treated with Ce6-ALUMSNs (0.5 μg/mL Ce6) for 12 h at pH 6.5, with exposure to NIR light of varying laser irradiation power densities (0.5–1.5 W/cm^2^) for 5 min. The four quadrants are defined as follows: annexin V–/PI– (bottom left), live cells; annexin V+/PI– (bottom right), early apoptotic cells; annexin V+/PI+ (top right), late apoptotic cells; and annexin V–/PI+ (top left), necrotic cells. (j) A summary of the incidence of early/late apoptosis and necrosis in the 4T1 cells treated with Ce6-ALUMSNs determined from the flow cytometry analysis of annexin V/PI staining in (i) (*n* = 4). *P < 0.05, **P < 0.01, ***P < 0.001, ****P < 0.0001 or nonsignificant (ns, *P* > 0.05) compared with controls.

In the presence of 980 nm laser light, treatment with Ce6-loaded ALUMSNs for 48–72 h at pH 7.4 did not adversely affect 4T1 cell viability (Figure 6a–c and Supporting Figure 10a–c), which is to be expected given the poor cell internalization of the nanospheres at physiological pH (Figure 5a,b). In contrast, exposure to Ce6-ALUMSNs for 48–72 h at pH 6.5 markedly reduced 4T1 cell viability, and the toxicity of the nanospheres scaled with PS concentration and laser power density/irradiation duration (Figure 6d–f and Supporting Figure 10d–f). The MTS assay results were supported by calcein AM/propidium iodide (PI) staining,^84,85^ which showed that treatment of the cells with Ce6-ALUMSNs at pH 6.5 in combination with 980 nm laser irradiation resulted in a marked decrease in live cells (calcein signal) and a concomitant increase in dead cells (PI signal) (Figure 6g). Together with the cell uptake experiments, the cell viability/toxicity assays confirm that ATRAM mediates both the pH-dependent cancer cell uptake and the associated NIR light-induced cytotoxicity of the coupled nanospheres.

To elucidate the mechanism of the cytotoxicity of Ce6-ALUMSNs, we carried out a number of complementary assays. First, we used the fluorescent ROS probe dihydroethidium (DHE) to detect intracellular ROS generation in 4T1 cells treated with PS-loaded nanospheres at pH 6.5 and subsequently irradiated with NIR light. The bright red DHE fluorescence signal observed in the confocal microscopy images reflects increased intracellular ROS levels upon NIR laser illumination (Figure 6h). Next, we used the fluorescent probe tetramethylrhodamine methyl ester (TMRM) to monitor mitochondrial membrane potential (ΔΨ_m_).^86^ Upon accumulation in active mitochondria, TMRM’s fluorescence intensity changes in response to alterations in ΔΨ_m_.^87−89^ Confocal microscopy images revealed that exposure of 4T1 cells to Ce6-loaded ALUMSNs and NIR irradiation dramatically decreased TMRM fluorescence, indicating substantial depolarization of ΔΨ_m_ (Supporting Figure 11), which agrees with reports that elevated intracellular ROS levels in PDT cause mitochondrial damage.^66,90,91^ Interestingly, hyperthermia has also been shown to induce opening of the pathological mitochondrial permeability transition pore and depolarize ΔΨ_m_.^92,93^ Finally, FITC-conjugated annexin V/PI staining and flow cytometry were used to detect apoptotic cells.^85,94^ Treatment of 4T1 cells with Ce6-ALUMSNs at pH 6.5, followed by 980 nm laser irradiation, resulted in >70% of the cells undergoing apoptosis (Figure 6i,j). Collectively, these results show that Ce6-ALUMSNs cause NIR light-induced toxicity selectively in malignant cells within a mildly acidic environment and suggest that this toxicity occurs via combined ROS generation and hyperthermia that lead to ΔΨ_m_ depolarization and apoptosis.

### Macrophage Recognition and Immunogenicity of ALUMSNs

To prevent opsonization and subsequent clearance by the mononuclear phagocyte system (MPS), a part of the innate immune system that consists of *monocytes*, *macrophages*, and *dendritic cells*,^95,96^ the nanospheres were “wrapped” in a lipid/PEG bilayer coat.^40^ PEG is commonly used as a “stealth polymer” in nanocarrier formulations to avoid opsonization and evade MPS clearance.^97^

Interaction of ALUMSNs with macrophages was assessed by first quantifying uptake of the nanospheres in differentiated human monocytic leukemia THP-1 cells, a well-established *in vitro* model of activated macrophages,^98^ using flow cytometry. While Ce6-loaded UMSNs were readily taken up by differentiated THP-1 cells at pH 7.4, negligible internalization of Ce6-ALUMSNs in the cells was detected under the same conditions (Supporting Figure 12a,b). Moreover, exposure to Ce6-UMSNs reduced viability of THP-1 cells and induced production of the inflammatory cytokines, tumor necrosis factor-α (TNF-α), and interleukin 1 beta (Il-1β), by the macrophages (Supporting Figure 12c,d). In contrast, no significant toxicity or TNF-α/Il-1β production was observed following treatment with Ce6-ALUMSNs (Supporting Figure 12c,d). These results demonstrate that ALUMSNs effectively escape recognition and uptake by macrophages, a property of the lipid/PEG-coated nanospheres that is critical to their capacity to effectively target tumors.

### Pharmacokinetics and Biodistribution of ALUMSNs

**Figure 7:**
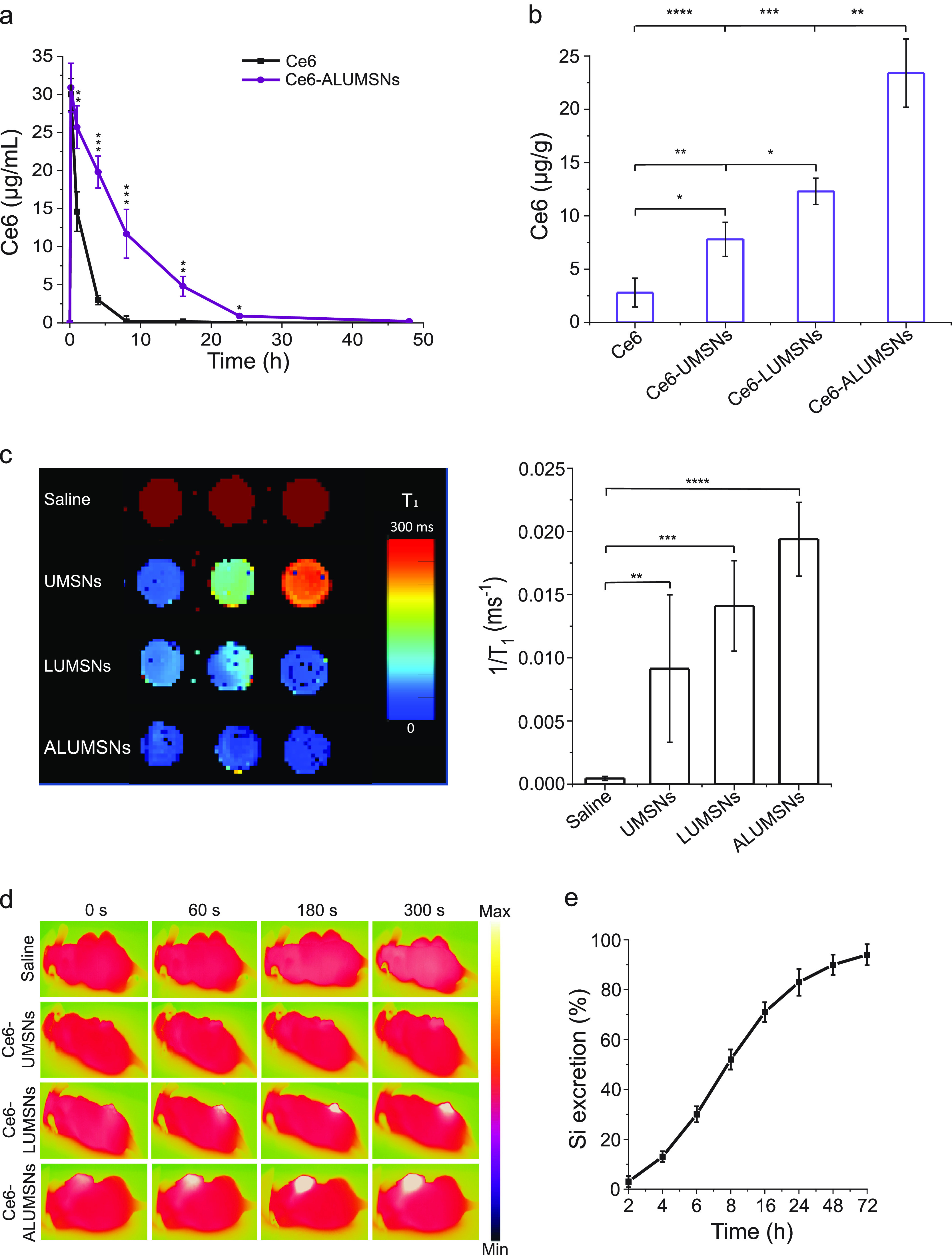
*In vivo* pharmacokinetics and tumor localization of ALUMSNs. (a) Concentration of Ce6 in plasma of test mice following a single *i.v*. injection of free Ce6 (2.5 mg/kg) or Ce6-loaded ALUMSNs (11 mg/kg nanospheres, 2.5 mg/kg Ce6) (*n* = 4 per group). (b) Concentration of Ce6 in 4T1 tumors in mice 8 h after a single *i.v*. injection of free Ce6 (2.5 mg/kg) or Ce6-loaded UMSNs, LUMSNs, or ALUMSNs (11 mg/kg nanospheres, 2.5 mg/kg Ce6) (*n* = 4 per group). Ce6 concentration in (a,b) was quantified using HPLC.^99^ (c) *T*_1_ maps (*left*) and relaxation rates (1/*T*_1_) (*right*) of 4T1 tumors isolated from mice 8 h following *i.v*. injection with saline or nanospheres (UMSNs, LUMSNs, or ALUMSNs; 11 mg/kg) (*n* = 3 per group). (d) Thermal imaging of 4T1 tumor-bearing mice upon NIR laser irradiation (1.0 W/cm^2^, 5 min) 8 h post *i.v*. injection with saline or Ce6-loaded UMSNs, LUMSNs, or ALUMSNs (11 mg/kg nanospheres, 2.5 mg/kg Ce6) (*n* = 4 per group). (e) Cumulative percentage of Si in urine and feces collected from test mice at various time points (2–72 h) post *i.v*. injection of ALUMSNs (11 mg/kg) (*n* = 4 per time point) determined by ICP-MS.^48^ *P < 0.05, **P < 0.01, ***P < 0.001, ****P < 0.0001 for comparisons with controls or among the different treatment groups.

Following intravenous injection of 4T1 tumor-bearing mice with Ce6, either in free form or encapsulated in nanospheres (UMSNs, LUMSNs, and ALUMSNs), blood was drawn at specific time points, and the concentration of PS in the samples was measured by high-performance liquid chromatography (HPLC).^99^ The *in vivo* circulation half-life of Ce6-ALUMSNs (*t*_1/2_ = 6.8 ± 2.1 h) was considerably longer than that of free Ce6 (*t*_1/2_ = 1.9 ± 0.9 h; Figure 7a). Furthermore, while free Ce6 was eliminated from the bloodstream in ∼8 h, the PS encapsulated in ALUMSNs persisted in the plasma up to 24 h post injection. The longer *in vivo* circulation time is expected to increase accumulation in target tumor tissue and, in turn, yield greater antitumor potency.^100^

To test this hypothesis, we performed the HPLC quantification of Ce6 in the 4T1 tumors. There was a much higher concentration of Ce6-loaded ALUMSNs (21.2 ± 5.0 μg Ce6/g of tumor tissue) in tumors compared to LUMSNs (12.5 ± 1.8 μg Ce6/g of tumor tissue), UMSNs (6.4 ± 2.5 μg Ce6/g of tumor tissue), or free Ce6 (6.3 ± 2.3 μg/g of tumor tissue) (Figure 7b). To ascertain whether ALUMSNs preferentially target tumors, we determined the biodistribution of the nanospheres using inductively coupled plasma mass spectrometry (ICP-MS).^48^ The advantage of ICP-MS is that it can accurately detect a wide range of elements simultaneously in a sample down to levels of ∼10 pg/mL. ICP-MS quantification of the Si content of tissue isolated from ALUMSN-treated mice showed significantly greater accumulation of the nanospheres in tumors compared to the heart, kidneys, liver, lungs, or spleen (Supporting Figure 13).

The tumor localization of the nanospheres was further investigated using magnetic resonance, thermal, and fluorescence imaging. *T*_1_ mapping revealed a stronger contrast enhancement effect (i.e., lower *T*_1_ relaxation time) in 4T1 tumors of mice treated with ALUMSNs compared to LUMSNs and UMSNs (Figure 7c). Similarly, thermal imaging following 980 nm laser irradiation (1.0 W/cm^2^) showed that Ce6-ALUMSNs induced a much more rapid and pronounced temperature increase in the tumors (from 36 to 55 °C within 5 min) compared to that of the other PS-loaded nanospheres (Figure 7d). Indeed, the photothermal response of the Ce6-ALUMSNs is comparable to that of other highly effective PTT nanomaterials,^61,62,101,102^ which underscores the high *in vivo* photothermal conversion efficiency and photostability of Ce6-ALUMSNs. Of relevance, hyperthermia not only serves to ablate cancer cells but has also been shown to increase intratumoral blood flow and enrich tumor oxygenation, which relieves tumoral microenvironment hypoxia and enhances PDT effects.^13,103,104^ Additionally, we assessed the tumor localization of Ce6-ALUMSNs using confocal fluorescence microscopy. Consistent with the ICP-MS results, we observed a noticeably higher fluorescence signal of the Ce6 cargo in sections of tumors compared to the vital organs (Supporting Figure 14). Taken together, these results illustrate that ALUMSNs effectively target tumors and facilitate multimodal—magnet resonance, thermal, and fluorescence—imagery of the cancerous tissue.

Finally, clearance of intravenously injected Ce6-loaded ALUMSNs was determined by measuring the Si content in the urine and feces of test mice at various time points (2–72 h) post injection using ICP-MS. Similar to other mesoporous silica-based nanoformulations,^105^ most of the ALUMSNs (∼95%) were excreted via urine and feces within 72 h following administration (Figure 7e), confirming the excellent biodegradability of the nanospheres.

### *In Vivo* Tumor Growth Inhibition by Ce6-Loaded ALUMSNs

Given the promising *in vitro* results of the Ce6-loaded ALUMSNs—potent and selective, NIR light-induced, anticancer activity (Figure 6) coupled with minimal interactions with serum proteins and macrophages (Supporting Figure 10)—as well as their effective tumor targeting (Figure 7), we next evaluated the antitumor efficacy of the nanospheres.

**Figure 8:**
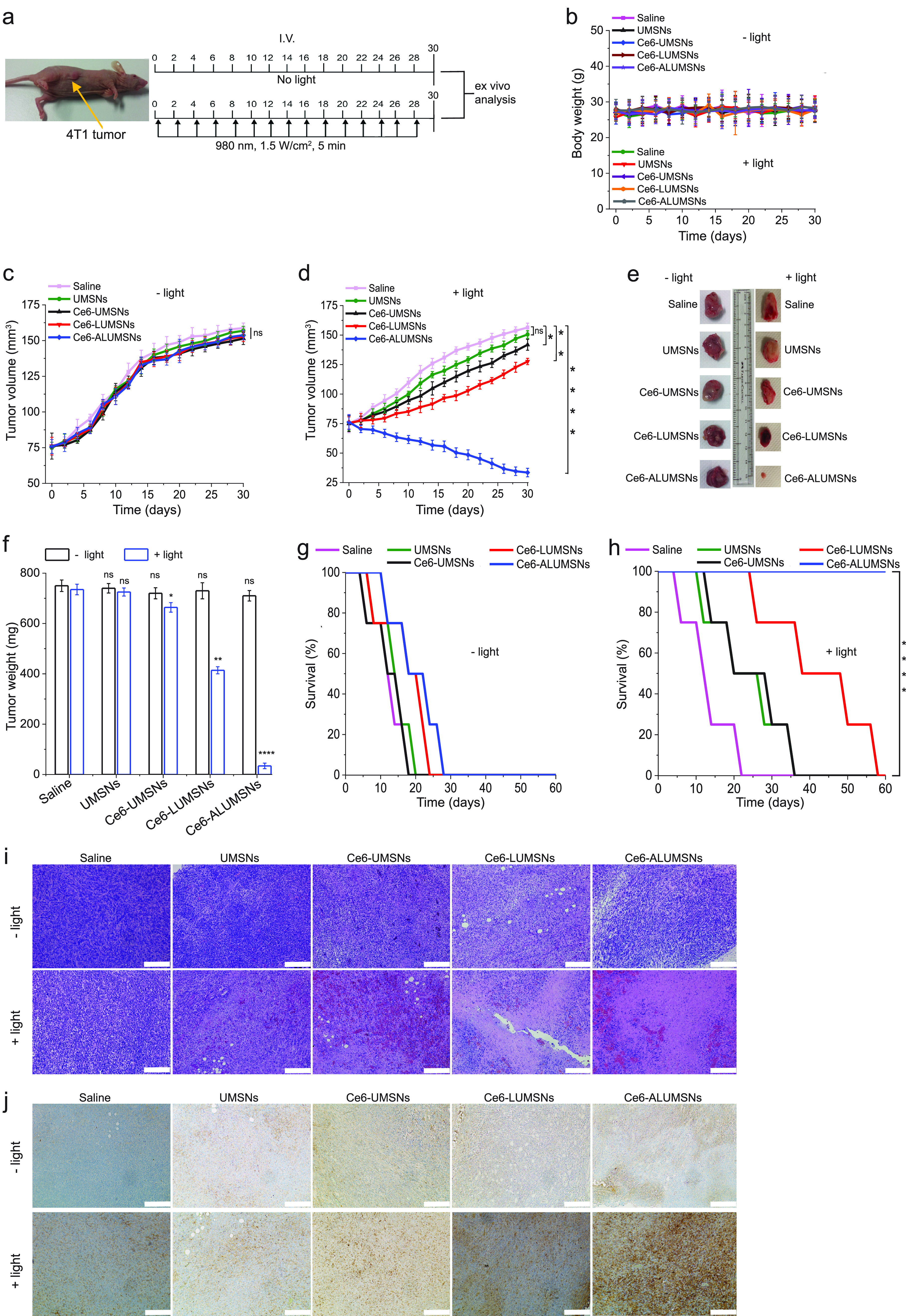
Inhibition of 4T1 tumor growth by Ce6-loaded ALUMSNs. (a) Design of the tumor reduction studies. Once the tumor volume reached ∼75 mm^3^, the mice were randomized into the different treatment groups (*n* = 16 per group), which were injected intravenously with saline, UMSNs (11 mg/kg) or Ce6-loaded UMSNs, LUMSNs, or ALUMSNs (11 mg/kg nanospheres, 2.5 mg/kg Ce6). Injections were done every other day for a total of 15 doses, with the first day of treatment marked as day 0. Within each treatment group, half of the mice were subjected to NIR laser irradiation (1.5 W/cm^2^, 5 min) at 8 h post injection. (b) Bodyweight changes of the 4T1 tumor-bearing mice in the different treatment groups in the absence (− light) or presence (+ light) of irradiation monitored for the duration of the experiment. (c,d) Tumor volume growth curves for the 4T1 tumors in the saline, UMSN, Ce6-UMSN, Ce6-LUMSN, and Ce6-ALUMSN treatment groups over 30 days of treatment in the absence (c) or presence (d) of NIR laser irradiation (*n* = 8 per group). (e,f) Tumor mass analysis for the saline, UMSN, Ce6-UMSN, Ce6-LUMSN, and Ce6-ALUMSN treatment groups. After 30 days of treatment, four mice per treatment group were sacrificed, and the tumor tissues were isolated and imaged (e) and subsequently measured weighed to determine the tumor mass (f). (g,h) Survival curves for the different treatment groups (saline, UMSNs, Ce6-UMSNs, Ce6-LUMSNs, and Ce6-ALUMSNs) over 60 days in the absence (g) or presence (h) of NIR laser irradiation (*n* = 4 per group). (i) H&E staining of 4T1 tumor sections from the different groups (saline, UMSNs, Ce6-UMSNs, Ce6-LUMSNs, and Ce6-ALUMSNs) after 30 days of treatment in the absence (− light) or presence (+ light) of NIR laser irradiation. (j) IHC images of 4T1 tumor sections stained with the cleaved caspase-3 antibody from the different groups after 30 days of treatment in the absence (− light) or presence (+ light) of NIR laser irradiation. Images shown in (i,j) are representative of tissue sections from four mice per treatment group; scale bar = 200 μm. *P < 0.05, **P < 0.01, ****P < 0.0001 or nonsignificant (ns, *P* > 0.05) for comparisons with controls.

Mice bearing 4T1 mammary carcinoma tumors were injected intravenously with UMSNs (11 mg/kg) or Ce6-loaded UMSNs, LUMSNs, or ALUMSNs (11 mg/kg nanospheres, 2.5 mg/kg Ce6), every 2 days for a total of 15 doses (Figure 8a). The Ce6 dose injected here is comparable to that used in other PDT-based cancer treatment studies.^106,107^ As expected, in the absence of 980 nm laser irradiation, none of the treatments had any significant effect on growth of the 4T1 tumors (Figure 8c,e,f) or survival of the mice (Figure 8g). In the presence of NIR laser irradiation, treatment with UMSNs yielded negligible anticancer effects, which were only modestly enhanced upon loading of the nanospheres with Ce6 (Figure 8d–f). A greater inhibition of tumor growth and a more pronounced lengthening of median survival time were observed in the Ce6-LUMSN treatment group (Figure 8d–f,h). However, treatment with Ce6-ALUMSNs yielded the greatest antitumor effects, decreasing the 4T1 tumors from an initial volume of 75 ± 7.8 to 33.5 ± 3.6 mm^3^ (Figure 8d) and the tumor mass to ∼5% of that of the controls (Figure 8e,f). Ce6-ALUMSNs also prolonged survival substantially compared to the controls and all of the other treatment groups over the duration of the experiment (Figure 8h).

Histological (hematoxylin and eosin (H&E)) staining corroborated the greater antitumor efficacy of Ce6-ALUMSNs compared to all other treatment groups (Figure 8i). Moreover, immunohistochemistry (IHC) analysis revealed increased levels of cleaved caspase-3, a crucial mediator of apoptosis,^108^ in tumor sections from the Ce6-ALUMSN treatment group (Figure 8j). To establish that NIR laser irradiation of Ce6-ALUMSNs not only causes localized hyperthermia (Figure 7d) but also leads to ROS generation in tumors *in vivo*, nanosphere-treated mice were injected intratumorally with DHE, with or without subsequent exposure to 980 nm laser light. A bright DHE signal, reflecting the presence of elevated intracellular ROS levels, was observed only in sections from the tumors subjected to NIR laser irradiation (Supporting Figure 15). These results are in agreement with the *in vitro* studies, which indicated that the NIR laser light-triggered cytotoxic effects of Ce6-ALUMSNs in cancer cells are due to combined PDT and PTT-mediated apoptosis (Figure 6).

Crucially, treatment with Ce6-ALUMSNs, in the absence or presence of NIR irradiation, did not have an adverse effect on the bodyweight of the mice (Figure 8b), and no apparent abnormalities or lesions were observed in H&E-stained sections of vital organs (heart, kidney, liver, lung, and spleen; Supporting Figure 16). Additionally, exposure to Ce6-ALUMSNs did not increase levels of cleaved caspase-3 in the vital organs nor did it significantly elevate concentrations of the inflammatory cytokines, TNF-α and Il-1β, in circulation (Supporting Figures 17 and 18). Finally, serum biochemical analysis also showed that treatment with Ce6-ALUMSNs did not significantly alter the levels of important health biomarkers, such as alanine aminotransferase (ALT), aspartate aminotransferase (AST), total bilirubin (TBILI), blood urea nitrogen (BUN), creatinine (CRE), triglycerides (TRG), and albumin (ALB) (Supporting Information Table 4), which further confirms the lack of systemic toxicity of the tumor-targeting nanospheres. Of note, our results are supported by the reported *in vivo* biocompatibility of a wide range of lanthanide-, yttrium-, and Bi_2_Se_3_-based nanomaterials for cancer therapy.^109−113^ Collectively, the *in vitro* and *in vivo* experiments clearly demonstrate that Ce6-loaded ALUMSNs potently shrink tumors *in vivo* via NIR irradiation-induced PDT and PTT, without adversely affecting healthy tissue, thereby markedly prolonging survival.

## Conclusions

Despite their promise as noninvasive light-based cancer treatments, PDT and PTT are currently beset by a number of issues that have hindered their clinical application. These include poor solubility, low stability, and lack of tumor specificity of many common PSs and PTAs.^5,7^ Moreover, the hypoxic microenvironment of solid tumors impairs PDT, since PSs require molecular oxygen to generate ROS,^9−11^ while hyperthermia-induced overexpression of heat shock proteins can attenuate the effects of PTT.^12−14^ Here, we developed multifunctional core–shell nanospheres that overcome these issues. The nanospheres are composed of a lanthanide- and PTA-doped upconversion core (NaYF_4_:Yb/Er/Gd,Bi_2_Se_3_); a PS (Ce6)-loaded mesoporous silica shell; and a lipid/PEG bilayer (DPPC/cholesterol/DSPE-PEG) coat, which is functionalized with the ATRAM peptide. The ATRAM-functionalized, lipid/PEG-coated upconversion mesoporous silica nanospheres (ALUMSNs) combine the following critical properties: (i) stable encapsulation of PTAs and PSs, which prevents their aggregation and protects them from premature degradation; (ii) minimal interactions with healthy tissue, serum proteins, and macrophages, leading to increased *in vivo* circulation half-life of the PTA and PS cargoes; (iii) efficient and specific internalization into cancer cells within a mildly acidic environment such as that of solid tumors; (iv) excitation by NIR light, which has greater tissue penetration, lower autofluorescence and reduced phototoxicity compared to visible light; (v) MRI (due to the presence of Gd in the core), NIR laser light-mediated thermal imaging, as well as fluorescence imaging capabilities; (vi) NIR laser light-induced PDT and PTT, the combination of which synergistically improves the efficacy of both phototherapies—PTT-induced hyperthermia increases local blood flow and leads to accumulation of molecular oxygen in tumor tissue and enhanced PDT, while ROS generated during PDT can inactivate heat shock proteins in cancer cells and increase their susceptibility to PTT^5,15^—resulting, in turn, in robust antitumor effects. Taken together, our studies underline the potential of the biocompatible and biodegradable ALUMSNs as a promising tumor-targeting nanoplatform that facilitates multimodal diagnostic imaging and potent combinatorial therapy.

## Experimental Section

### Cell Culture

Cell lines used in the study (acquired from American Type Culture Collection (ATCC)) underwent authentication and testing for mycoplasma contamination (Charles River Laboratories; Margate, UK). Murine breast cancer 4T1 cells (ATCC no. CRL-2539) and human monocytic leukemia THP-1 cells (ATCC no. TIB-202) were both cultured in RPMI 1640 medium supplemented with 10% FBS, 4 mM l-glutamine, 1 mM sodium pyruvate, and 1% penicillin/streptomycin (all from Sigma) at 37 °C in 5% CO_2_. Viability of the cells was monitored regularly during culturing using the Trypan Blue exclusion test on a Bio-Rad TC20 automated cell counter. Upon reaching ∼95% confluence, the cells were harvested using 0.25% trypsin-EDTA (Sigma) for use in the following experiments.

### Cancer Cell Uptake

For intracellular imaging, 4T1 cells were seeded at a density of 2 × 10^5^ cells/well in 500 μL of complete medium in four-chambered 35 mm glass bottom Cellview cell culture dishes (Greiner Bio-One; Monroe, NC, USA) and cultured for 24 h at 37 °C in 5% CO_2_. The medium was then replaced with fresh medium (pH 6.5 or 7.4) containing Ce6-ALUMSNs (0.5 μg/mL Ce6) and incubated at for a further 4 h. Finally, the media in the chambers was replaced once again with fresh media before the cells were imaged on an Olympus Fluoview FV-1000 confocal laser scanning microscope equipped with a 63× Plan-Apo/1.3 NA oil immersion objective with DIC capability. Image acquisition was done using the Olympus FV10-ASW Viewer software (version 4.2), and analysis was performed with the Fiji image processing software.^114^

To quantify cellular uptake, 4T1 cells were cultured in six-well plates (1 × 10^6^ cells/well) for 24 h at 37 °C in 5% CO_2_. Thereafter, the cells were incubated with Ce6-loaded LUMSNs or ALUMSNs (0.5 μg/mL Ce6) at pH 7.4 or 6.5 for 1 or 4 h. For the uptake pathway analysis, prior to addition of Ce6-ALUMSNs (0.5 μg/mL Ce6), the cells were pretreated for 1 h at pH 6.5 with 10 mM sodium azide/6 mM 2-deoxy-d-glucose in serum- and glucose-free medium or pretreated for 30 min at pH 6.5 with endocytosis inhibitors (10 μM chlorpromazine, 5 mM methyl-β-cyclodextrin, 5 μM filipin, or 5 μM amiloride) in serum-free medium. After addition of the nanospheres, the cells were maintained in the presence of inhibitors for a further 1 h at pH 6.5. The cells were then washed three times with ice-cold PBS to remove extracellular nanospheres, harvested by trypsinization, centrifuged (1000*g*, 5 min), and resuspended (500 μL ice-cold PBS with 10% FBS). Data was acquired by flow cytometry (10 000 cells/sample, gated on live cells by forward/side scatter and propidium iodide (PI) exclusion) on a FACSAria III cell sorter (BD Biosciences, San Jose, CA) with the Cy 5.5 filter, and analysis was performed using the FlowJo software (version 10.6).

### Cell Viability/Toxicity Assays

The cytotoxic effects of the nanospheres were probed using three complementary assays: (i) CellTiter 96 AQueous One Solution (MTS) assay, where the tetrazolium compound MTS (3-(4,5-dimethylthiazol-2-yl)-5-(3-carboxymethoxyphenyl)-2-(4-sulfophenyl)-2*H*-tetrazolium, inner salt) is reduced by intracellular dehydrogenases in live cells to a soluble formazan product;^82,83^ (ii) calcein AM/propidium iodide (PI) double staining, in which the cell-permeable nonfluorescent calcein AM is converted to fluorescent calcein by esterases in viable cells and the membrane-impermeant red-fluorescent PI, a nucleic acid-intercalating dye, is used as a counterstain;^84,85^ and (iii) Dead Cell Apoptosis assay, which detects exposed phosphatidylserine in apoptotic cells using Alexa 488-conjugated annexin V and simultaneously distinguishes between apoptosis and necrosis (by assessing plasma membrane integrity) using PI.^85,94^

The MTS assay was performed as previously described.^88,100,115^ Briefly, 4T1 cells were seeded at a density of 5 × 10^3^ cells/well in 100 μL of complete medium in standard 96-well plates. After culturing (37 °C, 5% CO_2_) for 24 h, the medium was replaced with fresh medium (pH 7.4 or 6.5) containing UMSNs or LUMSNs (5–100 μg/mL) or Ce6-ALUMSNs (0.05–5 μg/mL Ce6), and the cells were incubated for 48 h. The medium was then replaced once more with fresh medium to remove extracellular nanospheres, with or without subsequent exposure to NIR (980 nm) laser light of varying irradiation power densities (0.5–1.5 W/cm^2^) and durations (3.0 or 5.0 min). As a control, some cells were incubated at pH 7.4 or 6.5 for an additional 24 h after irradiation (for a total incubation time of 72 h). Thereafter, the medium was replaced with fresh medium containing 20 μL of MTS reagent, and the plates were incubated for a further 4 h. Finally, the absorbance of the formazan product (λ = 490 nm) of MTS reduction was measured on a BioTek Synergy H1MF Multi-Mode Microplate-Reader. Cells treated with carrier alone served as a control, while wells with medium alone were used as a blank. Cell viability was determined from the ratio of formazan absorbance of the nanosphere-treated cells to that of the carrier-treated controls.

For calcein AM/PI double staining, 4T1 cells were seeded at a density of 2 × 10^5^ cells/well in 500 μL of complete medium in four-chambered 35 mm glass bottom Cellview cell culture dishes and cultured (37 °C, 5% CO_2_) for 24 h. The cells were then treated with Ce6-ALUMSNs (0.5 μg/mL Ce6) for 12 h at pH 6.5, with or without subsequent exposure to NIR laser irradiation (980 nm, 1.0 W/cm^2^, 5 min) following replacement of the medium to remove extracellular nanospheres. After staining with 2 μM calcein AM and 1.5 μM PI for 30 min, the cells were imaged on an Olympus Fluoview FV-1000 confocal laser scanning microscope with a 63× Plan-Apo/1.3 NA oil immersion objective, and the images were processed using the Fiji software.

The Dead Cell Apoptosis assay was carried out as previously reported.^70,88,100^ 4T1 cells were seeded at a density of 1 × 10^6^ cells/well in six-well plates. After culturing (37 °C, 5% CO_2_) for 24 h, the medium was replaced with fresh medium containing Ce6-ALUMSNs (0.5 μg/mL Ce6), and the cells were incubated for a further 12 h at pH 6.5. Subsequently, the medium was replaced once more with fresh medium to remove extracellular nanospheres, and the cells were exposed to NIR laser light with varying irradiation power densities (0.5–1.5 W/cm^2^) for 5 min. The cells were then washed twice with ice-cold PBS, harvested by trypsinization, centrifuged (1000*g*, 5 min), and resuspended in 1× annexin-V-binding buffer (10 mM HEPES, 140 mM NaCl, 2.5 mM CaCl_2_, pH 7.4). Finally, the cells were stained with 5 μL Alexa 488-conjugated annexin V and 1 μg/mL PI for 30 min in the dark at ambient temperature. Data was acquired by flow cytometry (10 000 cells/sample) and analyzed using the FlowJo software.

### Intracellular Reactive Oxygen Species (ROS) Measurements

4T1 cells were seeded at a density of 2 × 10^5^ cells/well in 500 μL of medium in four-chambered 35 mm glass bottom Cellview cell culture dishes. After culturing (37 °C, 5% CO_2_) for 24 h, the cells were treated with Ce6-ALUMSNs (0.5 μg/mL Ce6) for 4 h at pH 6.5, with or without subsequent exposure to NIR laser irradiation (980 nm, 1.0 W/cm^2^, 5 min) following replacement of the medium to remove extracellular nanospheres. The medium was then replaced with fresh medium containing 5 μM ROS probe DHE, and the cells were incubated for an additional 30 min. Finally, the cells were imaged (Olympus Fluoview FV-1000 confocal laser scanning microscope), and the images were processed using the Fiji software.

### *In Vivo* Experiments

All animal experiments performed in this study were approved by the NYU Abu Dhabi Institutional Animal Care and Use Committee (NYUAD-IACUC; Protocol No. 21–0005) and adhered to established animal care and use guidelines.^116^ Experiments were performed on 6–8 week-old female BalbC nude mice (The Jackson Laboratory; Bar Harbor, ME, USA) that were maintained in air-filtered cages (20 °C, 50% humidity, 12 h light/dark cycle) and fed normal chow (Research Diets, New Brunswick, NJ) in the NYU Abu Dhabi Vivarium Facility.

To generate the tumors, 2 × 10^5^ viable 4T1 cells were injected subcutaneously into the right flank of the mice. Tumor dimensions were measured with high-precision calipers (Thermo Fisher), and tumor volume was calculated as follows: tumor volume (mm^3^) = (*W*^2^ × *L*)/2, where *W* and *L* are tumor width and length in mm, respectively. Once the tumor volume reached 75 mm^3^, experiments were performed as described in the following sections. Mice were monitored daily and euthanized once the tumor volume reached the burden defined by NYUAD-IACUC.

### Pharmacokinetics and Biodistribution

*In vivo* pharmacokinetics were measured using a previously published protocol.^70^ Test (non-tumor-bearing) mice were injected intravenously with a single dose of Ce6 or Ce6-ALUMSNs (2.5 mg/kg Ce6) (*n* = 4 per treatment group). Blood was drawn at various time points over 48 h from the saphenous vein, collected in K3-EDTA microcentrifuge tubes (Greiner Bio-One), and centrifuged (1500*g*, 5 min) to isolate the plasma. 100 μL of plasma was collected, spiked with Ce6 (1 μg/mL), and mixed with 100 μL of Tris buffer (1 M, pH 8). To extract the Ce6, the mixture was thrice diluted in 3 mL chloroform/methanol (9:1, v/v), vortexed (10 min), and centrifuged (2500*g*, 10 min). This was followed by collection and evaporation of the organic phase to dryness under a N_2_ stream. The dry residue was then dissolved in 60 μL of methanol and centrifuged (2500*g*, 10 min), and the supernatant was collected and filtered using a 0.2 μm syringe filter. Finally, 20 μL of supernatant was assayed by HPLC (Waters 2535 QGM HPLC).^99^

For ICP-MS analysis of biodistribution,^49^ 4T1 tumor-bearing mice were injected intravenously with a single dose of Ce6-ALUMSNs (11 mg/kg nanospheres, 2.5 mg/kg Ce6). At 8, 16, and 24 h post injection (*n* = 4 per time point), the mice were sacrificed, and tumors and vital organs (heart, kidneys, liver, lungs, and spleen) were harvested. Si concentrations in the tissues were then measured by ICP-MS against common standards (Agilent 7800 ICP-MS). ICP-MS was also used to quantify the rate of clearance of the tumor-targeting nanospheres from the treated test mice. At various time-points (2–72 h) post single *i.v*. injection (*n* = 4 per time point), urine and feces were collected, and Si concentration in the samples was determined by ICP-MS.^49^

Accumulation of the nanospheres in tumors *in vivo* was assessed using four techniques: (i) HPLC; (ii) MRI; (iii) thermal imaging; and (iv) fluorescence imaging. For HPLC-based quantification of *in vivo* tumor localization of the different nanospheres, 4T1 tumor-bearing mice were sacrificed, and the tumors were excised 8 h after a single *i.v*. injection of free Ce6 (2.5 mg/kg) or Ce6-loaded UMSNs, LUMSNs, or ALUMSNs (11 mg/kg nanospheres, 2.5 mg/kg Ce6) (*n* = 4 per group). The tumors were then homogenized in Tris buffer (1 M, pH 8), and Ce6 was extracted and quantified using the sample protocol for measuring concentrations of the PS in plasma described above. *Ex vivoT*_1_ mapping was performed using tumors excised from 4T1 tumor-bearing mice at 8 h post single *i.v*. injection with either saline or 11 mg/kg UMSNs, LUMSNs, or ALUMSNs (*n* = 3 per group). Imaging of the isolated tumors was done using the same procedure that was employed for the solution samples (Supporting Experimental Section). For *in vivo* thermal imaging, 4T1 tumor-bearing mice were injected intravenously with a single dose of saline or Ce6-loaded UMSNs, LUMSNs or ALUMSNs (11 mg/kg nanospheres, 2.5 mg/kg Ce6) (*n* = 4 per group). At 8 h post injection, the tumors were irradiated with NIR laser light (1.0 W/cm^2^, 5 min), and thermal images were captured using an Optris PI-640i infrared camera. For fluorescence-based assessment of tumor localization of the nanospheres, 4T1 tumor-bearing mice, fed on a chlorophyll-free diet, were given a single *i.v*. injection of saline or Ce6-ALUMSNs (11 mg/kg nanospheres and 2.5 mg/kg Ce6) (*n* = 4 per group). After 8 h, the mice were sacrificed, and the tumors and vital organs were harvested and cryosectioned onto slides. Finally, Ce6 fluorescence in the tissue sections was imaged using confocal laser scanning microscopy (Olympus Fluoview FV-1000).

### Tumor Growth Inhibition Studies

4T1-tumor bearing mice were randomly assigned to one of five treatment groups (*n* = 16 per treatment group), which were injected intravenously with saline, UMSNs (11 mg/g), or Ce6-loaded UMSNs, LUMSNs, or ALUMSNs (11 mg/kg nanospheres, 2.5 mg/kg Ce6). Injections were done every other day for a total of 15 doses, with the first day of treatment marked as day 0. Within each treatment group, half of the mice were subjected to NIR laser irradiation (1.5 W/cm^2^, 5 min) at 8 h post injection. Tumor volume and bodyweight were recorded every other day for the duration of treatment. After the 30 days of treatment, mice (*n* = 4 per group) were sacrificed to determine the tumor mass as well as for histological and immunohistochemical analyses of the tumors and vital organs (see Supporting Experimental Section).

Histological analysis using hematoxylin and eosin (H&E) staining of tissue sections was performed as previously described.^70^ Tumors and vital organs (heart, kidneys, liver, lungs, and spleen) were isolated, fixed in 10% formalin, embedded in paraffin, and sectioned into 4 μm slices using a Leica RM2235 microtome. The tissue sections were then dewaxed on microscope slides and stained with hematoxylin and eosin (H&E) using standard procedures.^117,118^ For immunohistochemistry (IHC) analysis, samples were prepared according to a published protocol.^119^ Briefly, tissue sections were treated with a heat-induced epitope retrieval solution (10 mM sodium citrate buffer, pH 6.0) for antigen recovery, blocked with 8% BSA, incubated overnight at 4 °C with the cleaved caspase-3 primary antibody (Asp175, 1:800), and then subjected to sequential incubations (45 min at room temperature) in biotinylated secondary antibody and streptavidin-horseradish peroxidase (Abcam). Finally, the signal was visualized upon incubation with the peroxidase substrate 3,3′-diaminobenzidine (Pierce DAB; Thermo Fisher). The sections were imaged on a NIKON LV100 upright microscope, and the images were processed by using the ECLIPSE LV software.

Detection of ROS in tumors *in vivo* was done according to a published protocol.^120^ Briefly, 4T1 tumor-bearing mice were given a single *i.v*. injection of Ce6-ALUMSNs (11 mg/kg of nanospheres, 2.5 mg/kg Ce6). At 8 h post injection, the tumors were directly injected with the ROS probe DHE (10 μM, 100 μL), with (+ light) or without (− light) subsequent exposure to NIR laser irradiation (1.5 W/cm^2^, 5 min) (*n* = 4 per group). The mice were then immediately sacrificed, and the tumors were excised, cryosectioned onto slides, and imaged using confocal laser scanning microscopy (Olympus Fluoview FV-1000).

To evaluate the systemic toxicity of the nanospheres, test mice were injected intravenously (every 2 days for a total of 15 doses) with saline, UMSNs (11 mg/g), or Ce6-loaded UMSNs, LUMSNs, or ALUMSNs (11 mg/kg nanospheres, 2.5 mg/kg Ce6) (*n* = 4 per group). At the end of the 30 days of treatment, the mice were sacrificed, and 0.75 mL of blood was drawn from the abdominal vena cava. Subsequently, the blood was centrifuged (1000*g*, 10 min) and the serum was carefully collected using a fine-bore pipet. Serum concentrations of inflammatory cytokines, TNF-α and IL-1β, were measured by using commercial ELISA kits. Serum biochemical analysis—including levels of liver and kidney function biomarkers—was performed on a Unicel DxC 600 Synchron Clinical System (Beckman Coulter; Brea, CA, USA).

### Statistical Analysis

To ensure unbiased results, all parts of the experiments—treatment, data acquisition, and data analysis—were performed by different investigators in a blinded manner. Sample sizes for the *in vivo* studies were determined using power calculations based on NYUAD-IACUC Protocol No. 21-0005. Error bars in this study represent the mean ± standard deviation of at least three independent replicates (i.e., *n* ≥ 3). Statistical analysis was performed by using GraphPad Prism (version 8.4.2). Statistical significance between two groups was determined using an unpaired *t* test, while among three or more groups, one-way analysis of variance (ANOVA) followed by Dunnett’s or Tukey’s *post hoc* test was used. *P* < 0.05 was considered to be statistically significant.

## Supporting Information Available

**Supporting Figures:** characterization of the core of the upconversion mesoporous silica nanospheres (UMSNs); characterization of the UMSNs; qualitative Jablonski diagram illustrating the upconversion process; size distribution analysis of lipid/PEG-coated UMSNs (LUMSNs); quantitative proteomic analysis of serum protein adsorption to the surface of LUMSNs; photothermal response of Ce6-loaded LUMSNs at lower near-infrared (NIR) laser power densities; long-term colloidal stability of ATRAM-functionalized LUMSNs (ALUMSNs) at acidic tumoral pH; pH-dependent cellular internalization of ALUMSNs; cytotoxicity of the nanospheres in the absence of NIR laser irradiation; MTS cell viability measurements 24 h following NIR laser irradiation; mitochondrial membrane potential (ΔΨ_m_) depolarization by the nanospheres; macrophage recognition and immunogenicity of the nanospheres; determination of *in vivo* biodistribution of ALUMSNs by inductively coupled plasma mass spectrometry (ICP-MS); fluorescence-based assessment of tumor localization of Ce6-loaded ALUMSNs; detection of reactive oxygen species (ROS) generation in tumors following treatment with Ce6-loaded ALUMSNs and subsequent NIR laser irradiation; histological analysis of vital organs following treatment with the nanospheres; immunohistochemistry (IHC) analysis of vital organs following treatment with Ce6-loaded ALUMSNs; quantification of inflammatory cytokines in serum following treatment with the nanospheres. **Supporting Information Tables:** summary of hydrodynamic diameters and zeta potentials of UMSNs, LUMSNs, and ALUMSNs; Ce6 loading capacity of UMSNs; proteins corresponding to the UniProt Knowledgebase (UniProtKB) accession numbers shown in Supporting Figure 5; serum biochemistry profile of Ce6-loaded ALUMSN-treated mice. **Supporting Experimental Section:** reagents; synthesis of the upconversion core of the nanospheres; synthesis of the upconversion mesoporous silica nanospheres (UMSNs), lipid/PEG-coated UMSNs (LUMSNs), and ATRAM-functionalized LUMSNs (ALUMSNs); characterization of the nanospheres; *T*_1_ relaxation measurements; photodynamic and photothermal response; NIR light-triggered cargo release; quantitative proteomics; mitochondrial membrane potential (ΔΨ_m_) measurements; macrophage recognition, toxicity and immunogenicity (PDF)

## Notes

An earlier version of this manuscript was previously submitted to an online preprint server. Palanikumar, L.; Kalmouni, M.; Houhou, T.; Abdullah, O.; Ali, L.; Pasricha, R.; Thomas, S.; Afzal, A. J.; Barrera, F. N.; Magzoub, M. pH-responsive upconversion mesoporous silica nanospheres for combined multimodal diagnostic imaging and targeted photodynamic and photothermal cancer therapy. *bioRxiv*, 2023. 10.1101/2023.05.22.541491 (accessed May 24, 2023).
