# Supplementary material for: pH-Responsive Upconversion Mesoporous Silica Nanospheres for Combined Multimodal Diagnostic Imaging and Targeted Photodynamic and Photothermal Cancer Therapy
Source: ACS Nano. 2023 Sep 13;17(19):18979–99. doi: 10.1021/acsnano.3c04564 (PMC10569106; doi:10.1021/acsnano.3c04564)
Supplement: Supplementary file 1 — nn3c04564_si_001.pdf [file nn3c04564_si_001.pdf]

SUPPORTING INFORMATION

pH-Responsive Upconversion Mesoporous Silica Nanospheres for Combined Multimodal Diagnostic Imaging and Targeted Photodynamic and Photothermal Cancer Therapy

L. Palanikumar,[*,†] Mona Kalmouni,[†] Tatiana Houhou,[†] Osama Abdullah,[‡] Liaqat Ali,[‡] Renu Pasricha,[‡] Rainer Straubinger,[‡] Sneha Thomas,[‡] Ahmed Jawaad Afzal,[†] Francisco N. Barrera,[§] and Mazin Magzoub[*,†]

[†]Biology Program, Division of Science, New York University Abu Dhabi, P.O. Box 129188, Saadiyat Island, Abu Dhabi, United Arab Emirates

[‡]Core Technology Platforms, New York University Abu Dhabi, P.O. Box 129188, Saadiyat Island, Abu Dhabi, United Arab Emirates

[§]Department of Biochemistry & Cellular and Molecular Biology, University of Tennessee Knoxville, Knoxville, TN 37996, United States

[*]Corresponding Authors

Mazin Magzoub – Biology Program, Division of Science, New York University Abu Dhabi, P.O. Box 129188, Saadiyat Island, Abu Dhabi, United Arab Emirates. Email: mazin.magzoub@nyu.edu

L. Palanikumar – Biology Program, Division of Science, New York University Abu Dhabi, P.O. Box 129188, Saadiyat Island, Abu Dhabi, United Arab Emirates. Email: pl105@nyu.edu

TABLE OF CONTENTS

SUPPORTING FIGURES

Supporting Figure 1. Characterization of the core of the upconversion mesoporous silica nanospheres (UMSNs)

Supporting Figure 2. Characterization of the UMSNs

Supporting Figure 3. Qualitative Jablonski diagram illustrating the upconversion process

Supporting Figure 4. Size distribution analysis of lipid/PEG-coated UMSNs (LUMSNs)

Supporting Figure 5. Quantitative proteomic analysis of serum protein adsorption to the surface of LUMSNs

Supporting Figure 6. Photothermal response of Ce6-loaded LUMSNs at lower near-infrared (NIR) laser power densities

Supporting Figure 7. Long-term colloidal stability of ATRAM-functionalized LUMSNs (ALUMSNs) at acidic tumoral pH

Supporting Figure 8. pH-dependent cellular internalization of ALUMSNs

Supporting Figure 9. Cytotoxicity of the nanospheres in the absence of NIR laser irradiation

Supporting Figure 10. MTS cell viability measurements 24 h following NIR laser irradiation

Supporting Figure 11. Mitochondrial membrane potential ($\Delta\Psi_m$) depolarization by the nanospheres

Supporting Figure 12. Macrophage recognition and immunogenicity of the nanospheres

Supporting Figure 13. Determination of *in vivo* biodistribution of ALUMSNs by inductively coupled plasma mass spectrometry (ICP-MS)

Supporting Figure 14. Fluorescence-based assessment of tumor localization of Ce6-loaded ALUMSNs

Supporting Figure 15. Detection of reactive oxygen species (ROS) generation in tumors following treatment with Ce6-loaded ALUMSNs and subsequent NIR laser irradiation

Supporting Figure 16. Histological analysis of vital organs following treatment with the nanospheres

Supporting Figure 17. Immunohistochemistry (IHC) analysis of vital organs following treatment with Ce6-loaded ALUMSNs

Supporting Figure 18. Quantification of inflammatory cytokines in circulation following treatment with the nanospheres

SUPPORTING INFORMATION TABLES

Table 1. Summary of hydrodynamic diameters and zeta potentials of UMSNs, LUMSNs and ALUMSNs
Table 2. Ce6 loading capacity of UMSNs
Table 3. Proteins corresponding to the UniProt Knowledgebase (UniProtKB) accession numbers shown in Supporting Figure 5
Table 4. Serum biochemistry profile of Ce6-loaded ALUMSN-treated test mice

SUPPORTING EXPERIMENTAL SECTION

Section 1. Reagents
Section 2. Synthesis of the Upconversion Core of the Nanospheres
Section 3. Synthesis of the Upconversion Mesoporous Silica Nanospheres (UMSNs)
Section 4. Synthesis of Lipid/PEG-Coated UMSNs (LUMSNs)
Section 5. Synthesis of ATRAM-Functionalized LUMSNs (ALUMSNs)
Section 6. Characterization of the Nanospheres
Section 7. T_1 Relaxation Measurements
Section 8. Photodynamic and Photothermal Response
Section 9. NIR Light-Triggered Cargo Release
Section 10. Quantitative Proteomics
Section 11. Mitochondrial Membrane Potential ($\Delta\Psi_m$) Measurements
Section 12. Macrophage Recognition, Toxicity and Immunogenicity

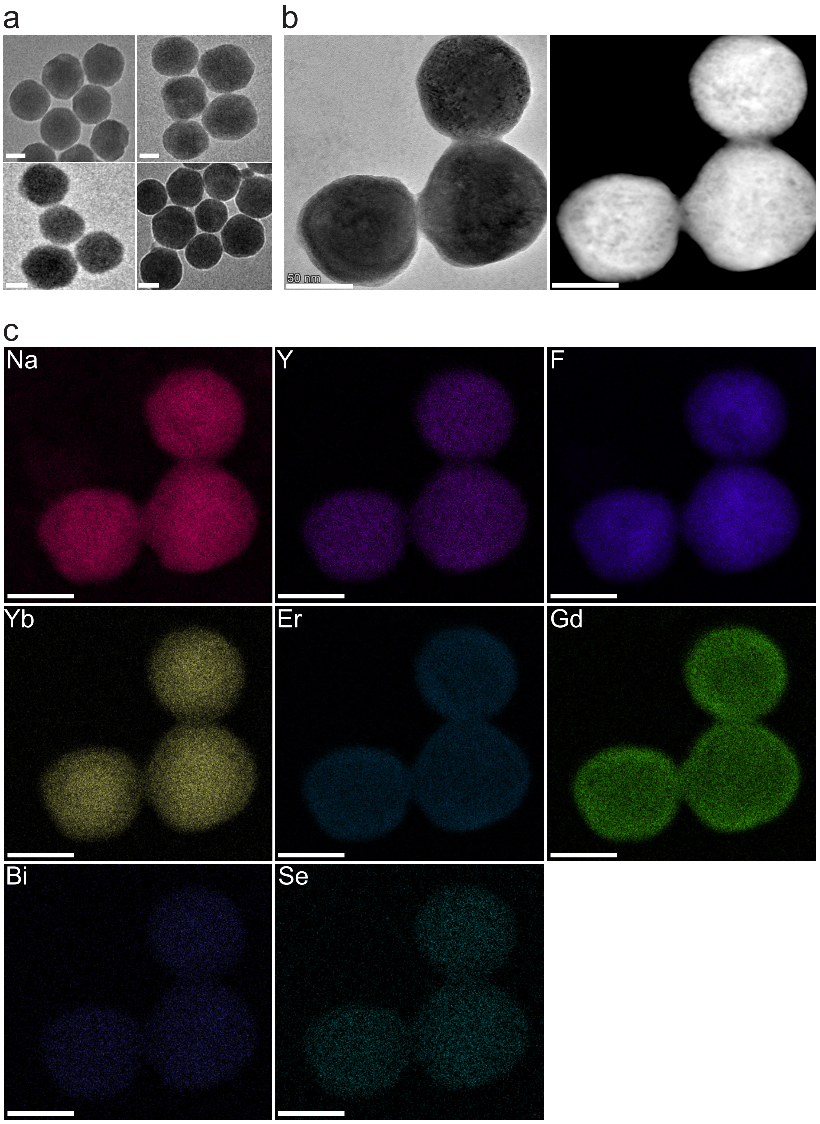

Supporting Figure 1. Characterization of the core of the upconversion mesoporous silica nanospheres (UMSNs). (**a**) Transmission electron microscopy (TEM) images of the upconversion cores ($NaYF_4$:Yb/Er/Gd,Bi_2Se_3) of UMSNs. Each of the four quadrants shows cores from a different preparation. (**b**) Higher magnification TEM (*left*) and scanning transmission electron microscopy (STEM) (*right*) images of the upconversion cores of UMSNs. (**c**) Scanning transmission electron microscopy-energy dispersive X-ray spectroscopy (STEM-EDS) mapping of the composition of the upconversion cores in (**b**). Scale bar = 50 nm.

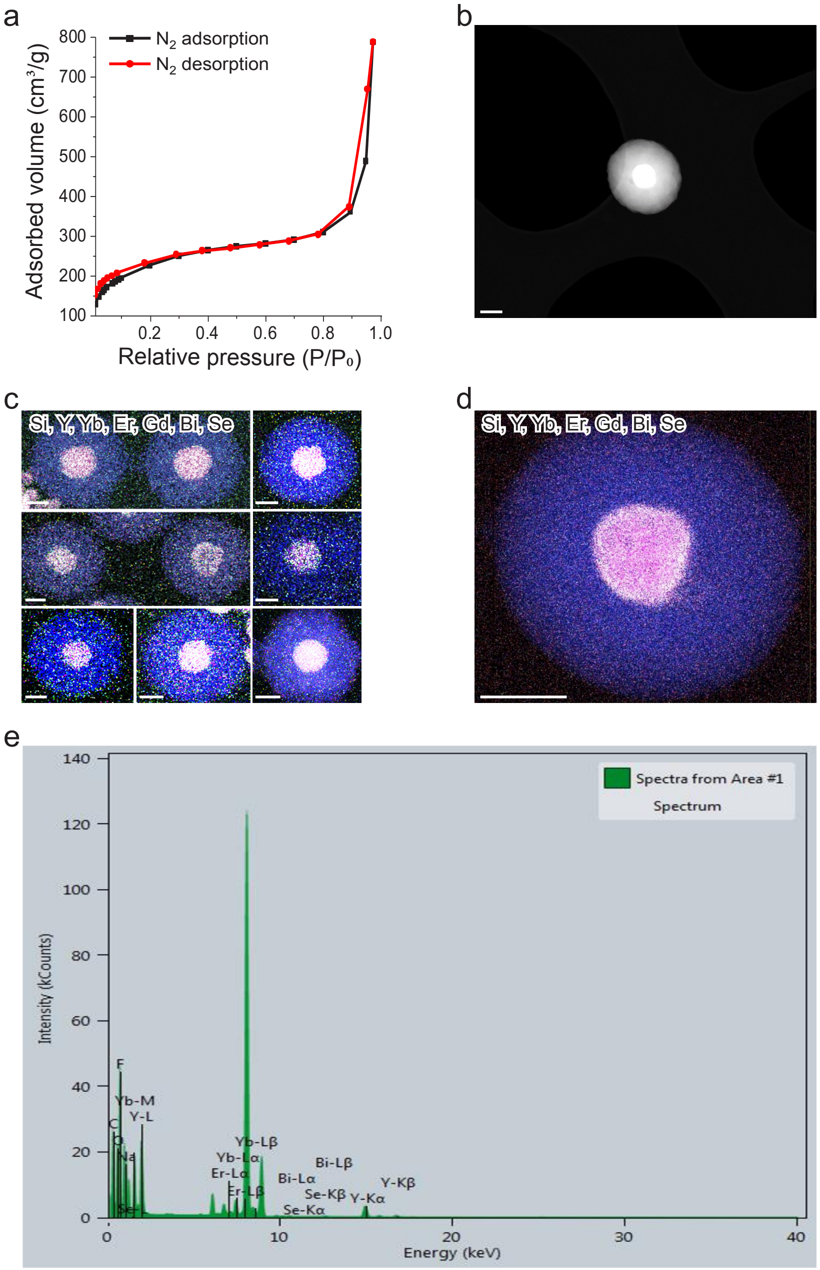

Supporting Figure 2. Characterization of the upconversion mesoporous silica nanospheres (UMSNs). (**a**) N_2 adsorption-desorption isotherms of UMSNs. (**b**) High-angle annular dark-field scanning transmission electron microscopy (HAAD-STEM) image of a representative UMSN. (**c**) Scanning transmission electron microscopy-energy dispersive X-ray spectroscopy (STEM-EDS) mapping of UMSNs. The UMSNs in the individual panels are from independent preparations. (**d**) Higher resolution STEM-EDS mapping of a representative UMSN. (**e**) EDS spectrum of the UMSN in (**d**). Scale bar in (**b–d**) = 50 nm.

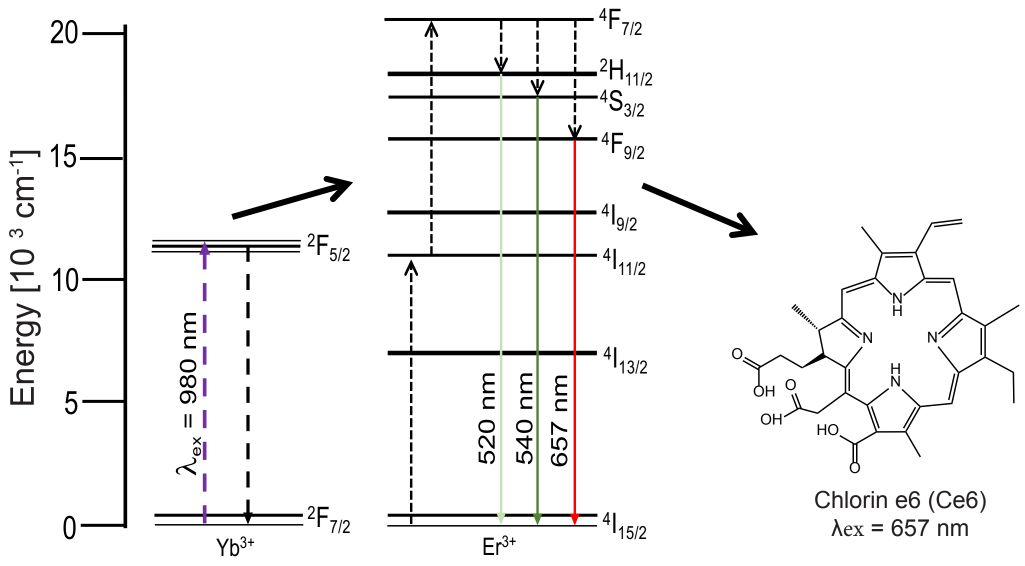

Supporting Figure 3. Qualitative Jablonski diagram illustrating the upconversion process. The trivalent ytterbium ion (Yb^{3+}) effectively absorbs near-infrared (NIR) light (980 nm) and transfers the energy to the erbium ion (Er^{3+}), resulting in multiple higher energy visible light emissions from Er^{3+}, the strongest of which are in the green and the red.[1] The red emission (657 nm) from Er^{3+} excites the photosensitizer chlorin e6 (Ce6), which then transfers its energy to molecular oxygen, converting the latter from its ground state to its cytotoxic singlet state ($^1O_2$).[1]

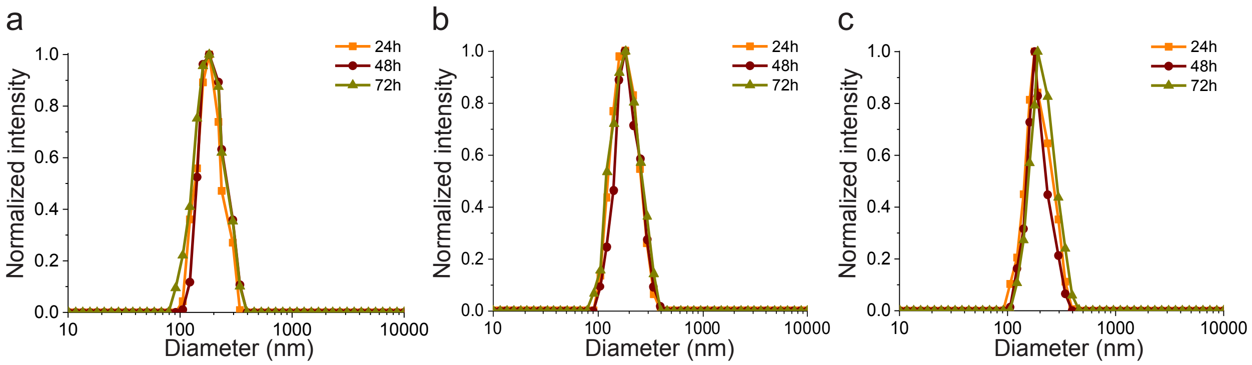

Supporting Figure 4. Size distribution analysis of lipid/PEG-coated UMSNs (LUMSNs). Dynamic light scattering (DLS) measurements of size of LUMSNs in 10 mM phosphate buffer (pH 7.4) (**a**), 50 mM sodium acetate buffer (pH 5.5) (**b**), and complete cell culture medium (RPMI 1640 containing 10% fetal bovine serum (FBS), pH 7.4) (**c**), over 72 h.

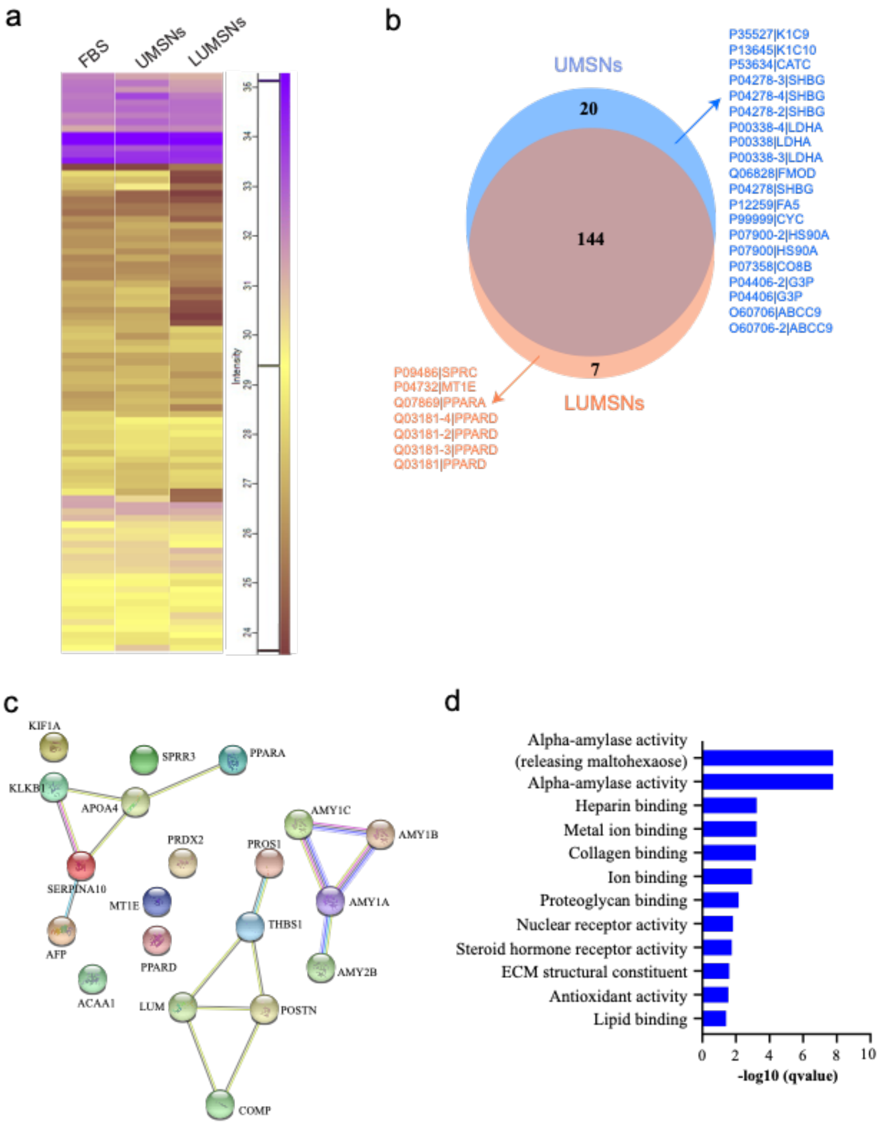

Supporting Figure 5. Quantitative proteomic analysis of serum protein adsorption to the surface of LUMSNs. (**a**) Heat map representation of identified serum proteins in the control fetal bovine serum (FBS) sample, and adsorbed to the surface of UMSNs and LUMSNs following incubation in complete cell culture medium (RPMI 1640 containing 10% FBS, pH 7.4) for 72 h. The digests for both the control and nanosphere samples were analyzed by liquid chromatography tandem mass spectrometry (LC-MS/MS) and protein abundance was determined using label-free quantification (LFQ).[2] As shown in the color scale bar, the purple and yellow (gold) colors indicate high and low LFQ intensities (log2 (LFQ)), respectively, while dark brown indicates that the protein concentration is below the detection limit. The proteins corresponding to the UniProt Knowledgebase (UniProtKB)[3] accession numbers shown in the figure are given in Supporting Information Table 3. (**b**) Venn diagram delineating adsorption of the 144 most abundant serum proteins to the surface of LUMSNs compared to UMSNs. (**c**) Protein–protein interaction (PPI) network map of the serum proteins depleted or absent from the surface of LUMSNs compared to UMSNs. (**d**) Gene ontology analysis for serum proteins depleted or absent from the surface of LUMSNs compared to UMSNs.

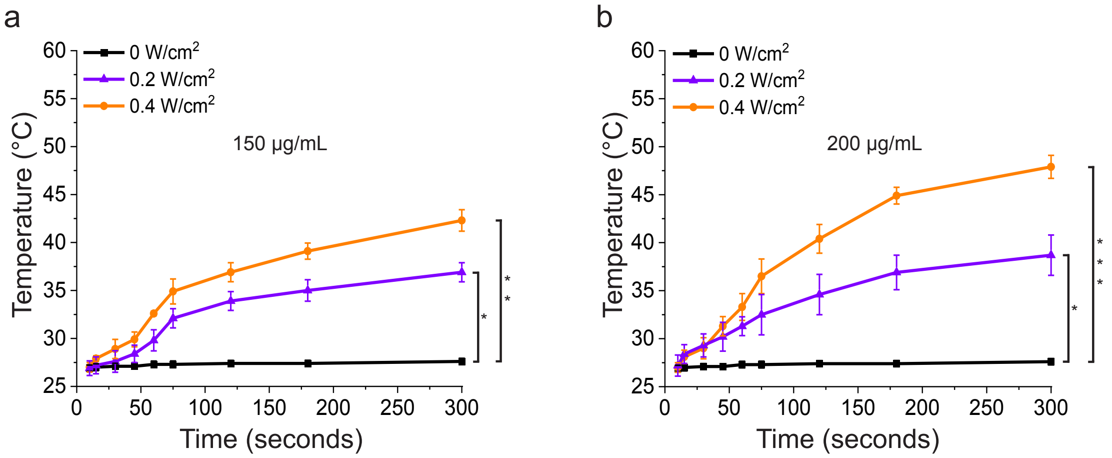

Supporting Figure 6. Photothermal response of Ce6-loaded LUMSNs at lower near-infrared (NIR) laser power densities. Temperature increases following NIR laser irradiation (0.2–0.4 W/cm^2, 5 min) of Ce6-LUMSNs at nanosphere concentrations of 150 (**a**) and 200 µg/mL (**b**) in 10 mM phosphate buffer (pH 7.4). **P* < 0.05, ***P* < 0.01, ****P* < 0.001 for comparisons between the samples.

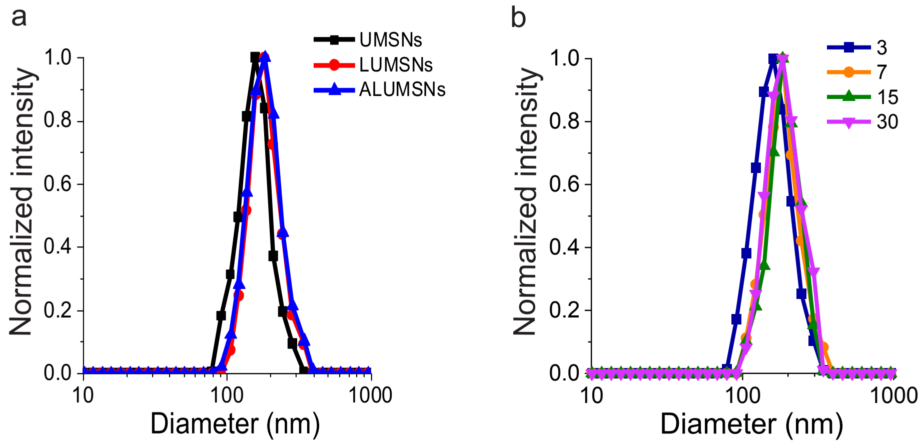

Supporting Figure 7. Long-term colloidal stability of ATRAM-functionalized LUMSNs (ALUMSNs) at acidic tumoral pH. (**a**) Size analysis for UMSNs, LUMSNs and ALUMSNs in complete cell culture medium (RPMI 1640 containing 10% FBS, pH 6.5) using dynamic light scattering (DLS). (**b**) Size analysis for ALUMSNs in complete cell culture medium (RPMI 1640 containing 10% FBS, pH 6.5) over 30 days.

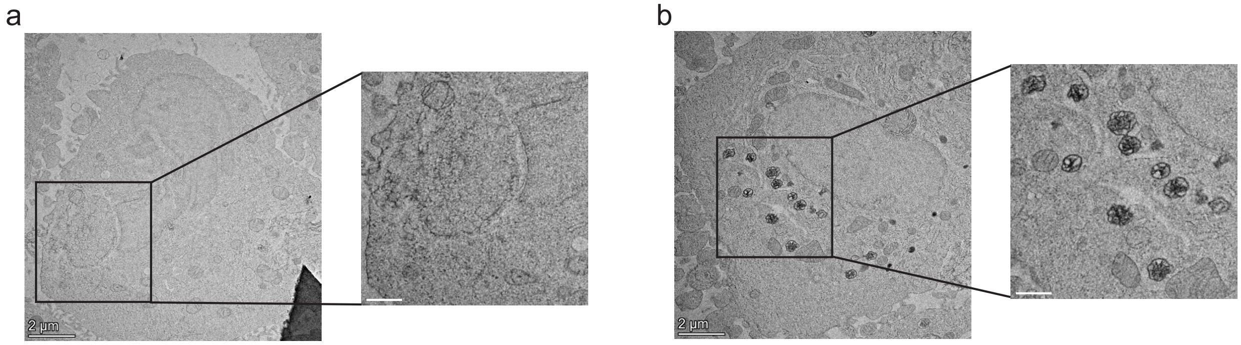

Supporting Figure 8. pH-dependent cellular internalization of ALUMSNs. Transmission electron microscopy images of 4T1 cells incubated with 3 µg/mL ALUMSNs for 4 h at pH 7.4 (**a**) vs 6.5 (**b**). Images on the right show magnified views of the marked areas of the images on the left. Scale bar = 2 µm.

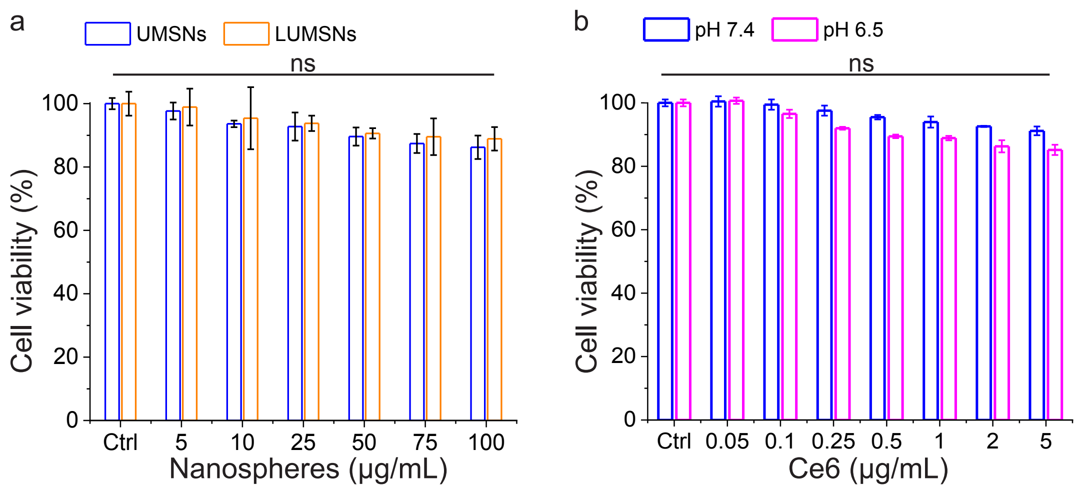

Supporting Figure 9. Cytotoxicity of the nanospheres in the absence of NIR laser irradiation. Cell viability of 4T1 cells treated with increasing concentrations of UMSNs or LUMSNs at physiological pH (**a**), or increasing concentrations of Ce6-loaded ALUMSNs (Ce6-ALUMSNs) at pH 7.4 or 6.5 (**b**), for 48 h. Cell viability was measured using the MTS assay,[4,5] with the % viability determined from the ratio of the absorbance of the treated cells to the control cells ($n = 4$). ns, non-significant ($P > 0.05$) for comparisons with vehicle-treated controls.

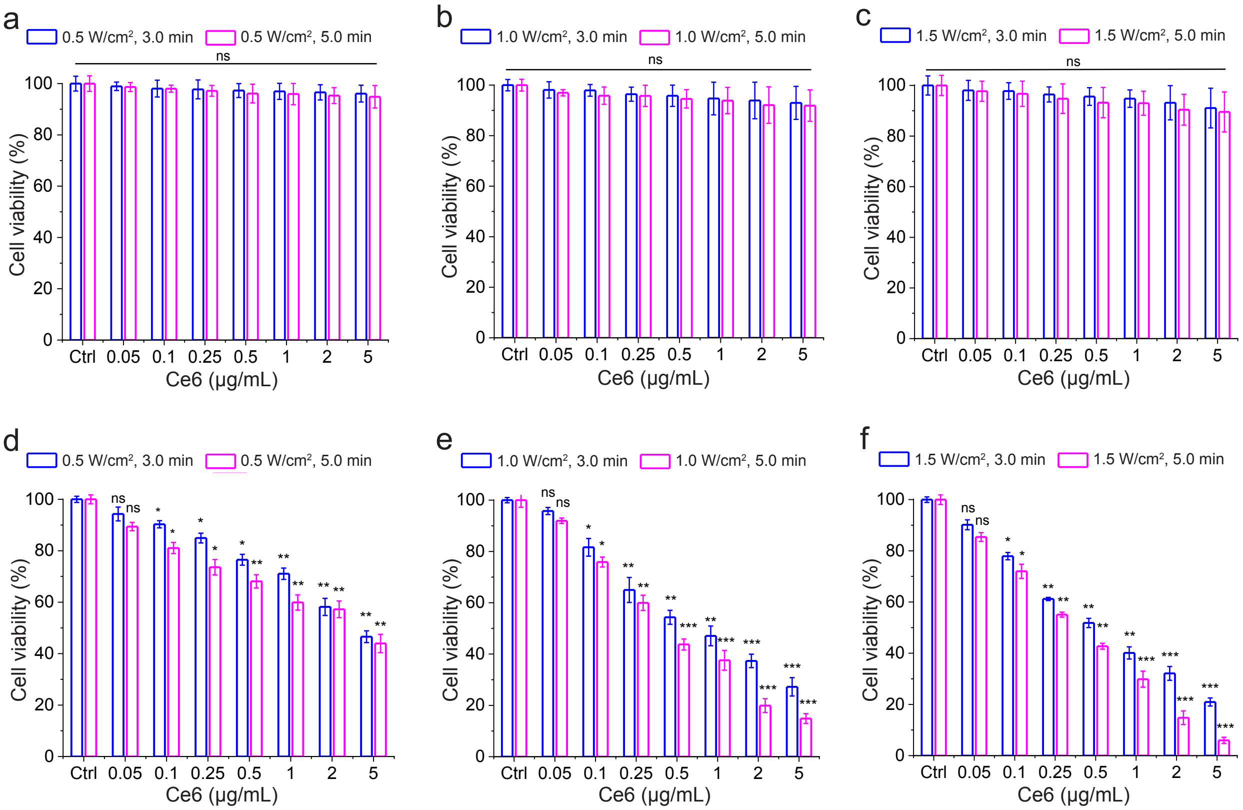

Supporting Figure 10. MTS cell viability measurements 24 h following NIR laser irradiation. Cell viability of 4T1 cells treated with Ce6-ALUMSNs (0.05–5 μg/mL Ce6) at pH 7.4 (**a**–**c**) or 6.5 (**d**–**f**). 4T1 cells were incubated with the nanospheres for 48 h. The medium was then replaced with fresh medium to remove extracellular nanospheres, and the cells were exposed to NIR laser light with varying irradiation power densities (0.5–1.5 W/cm^2) and durations (3 or 5 min). Subsequently, the cells were incubated for a further 24 h (for a total incubation time of 72 h) before cell viability was measured using the MTS assay ($n = 4$). *$P < 0.05$, **$P < 0.01$, ***$P < 0.001$, or non-significant (ns, $P > 0.05$) compared with controls.

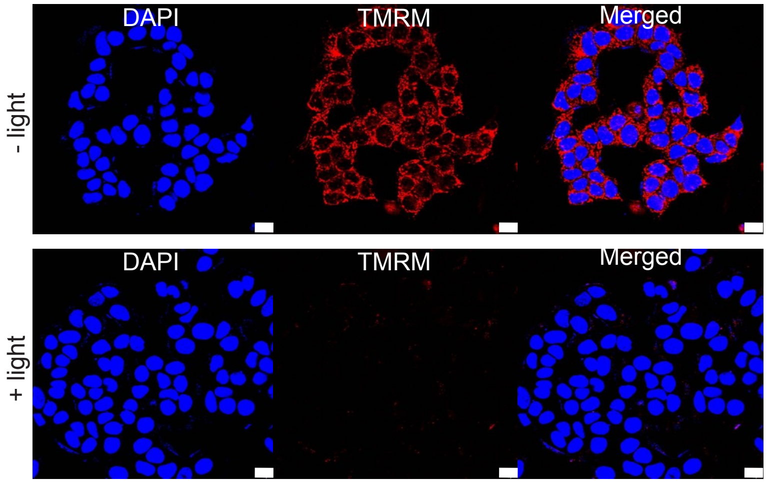

Supporting Figure 11. Mitochondrial membrane potential ($\Delta\Psi_m$) depolarization by the nanospheres. Confocal laser scanning microscopy images of tetramethylrhodamine methyl ester (TMRM, $\Delta\Psi_m$ reporter dye)[6] staining of 4T1 cells treated with Ce6-loaded ALUMSNs (0.5 µg/mL Ce6) for 4 h at pH 6.5 in the absence (- light) or presence (+ light) of irradiation with NIR laser (980 nm, 1.0 W/cm^2, 5 min). Scale bar = 10 µm.

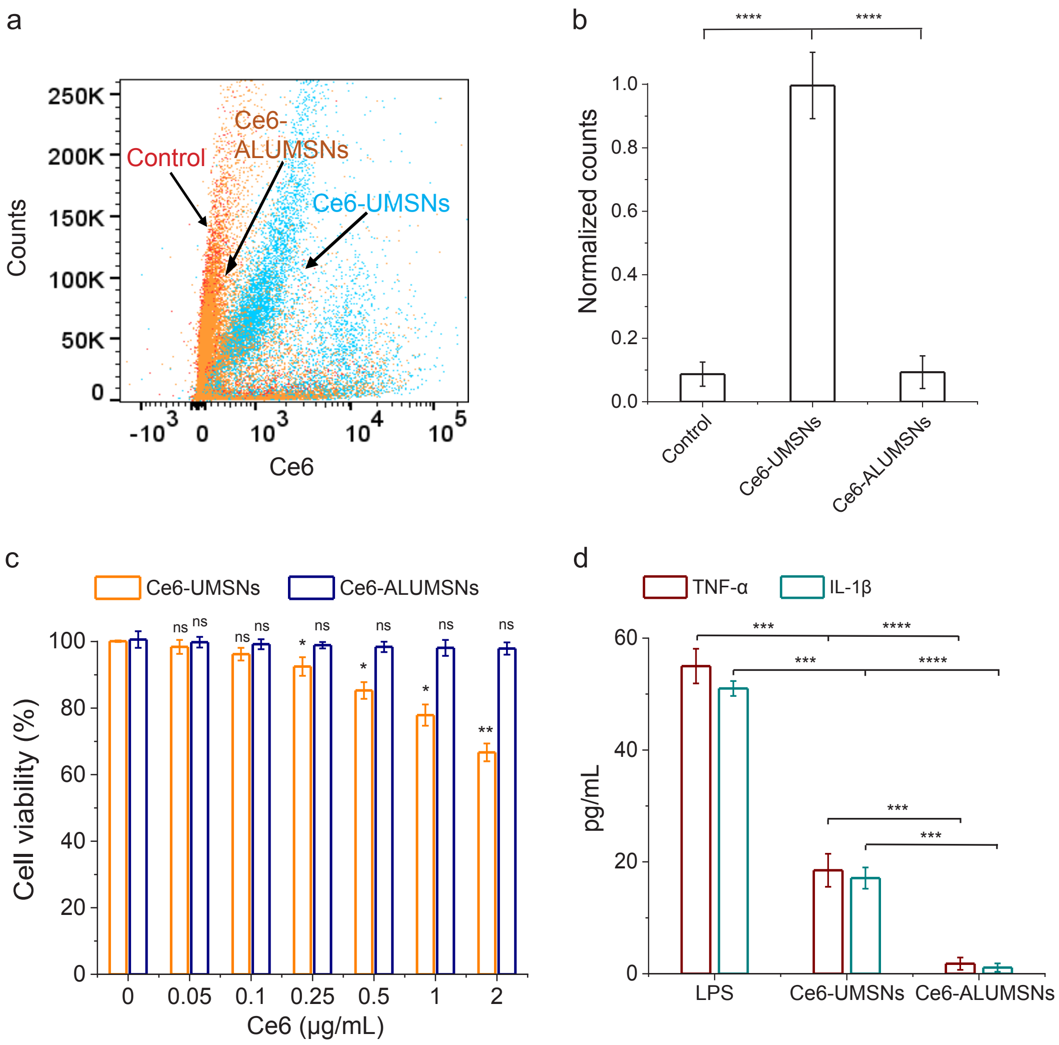

Supporting Figure 12. Macrophage recognition and immunogenicity of the nanospheres. (**a**, **b**) Flow cytometry analysis of differentiated THP-1 cells, which are widely used model of monocyte/macrophage activation,[7] that were either untreated (control), or treated with Ce6-UMSNs or Ce6-ALUMSNs (0.5 μg/mL Ce6) for 4 h at pH 7.4 (**a**); quantification of cellular uptake of the nanospheres from the flow cytometry analysis ($n = 4$) (**b**). (**c**) Cell viability of differentiated THP-1 cells treated with Ce6-UMSNs or Ce6-ALUMSNs for 48 h at pH 7.4. Cell viability was assessed using the MTS assay ($n = 4$). (**d**) Release of inflammatory cytokines, tumor necrosis factor-alpha (TNF-α) and interleukin-1 beta (IL-1β), by differentiated THP-1 cells exposed to Ce6-UMSNs or Ce6-ALUMSNs (0.5 μg/mL Ce6) for 24 h at pH 7.4. Cells treated with the macrophage activator lipopolysaccharide (LPS)[8] were used as a positive control for inflammation. TNF-α and IL-1β levels in the culture medium were assayed using a commercial ELISA kit ($n = 4$). *$P < 0.05$, **$P < 0.01$, ***$P < 0.001$, ****$P < 0.0001$ or non-significant (ns, $P > 0.05$) compared with controls.

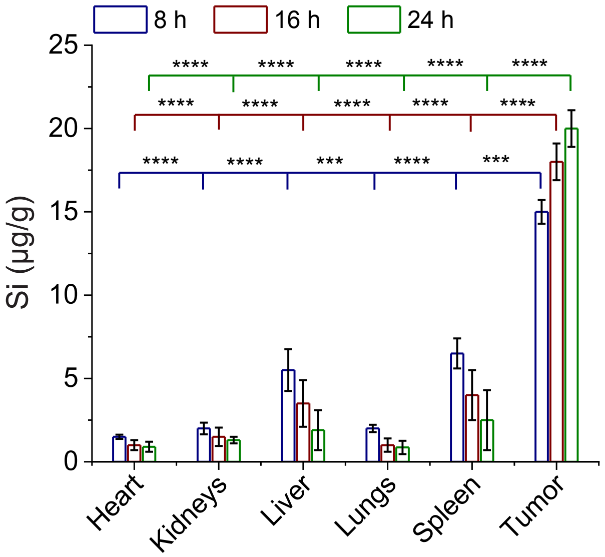

Supporting Figure 13. Determination of *in vivo* biodistribution of ALUMSNs by inductively coupled plasma mass spectrometry (ICP-MS). Quantification of Si in tumors and vital organs (heart, kidneys, liver, lungs, and spleen), isolated from 4T1 tumor-bearing mice at 8, 16 and 24 h after a single *i.v.* injection of ALUMSNs (11 mg/kg nanospheres), by ICP-MS[9] ($n = 4$). ***$P < 0.001$, ****$P < 0.0001$ for comparisons amongst the samples.

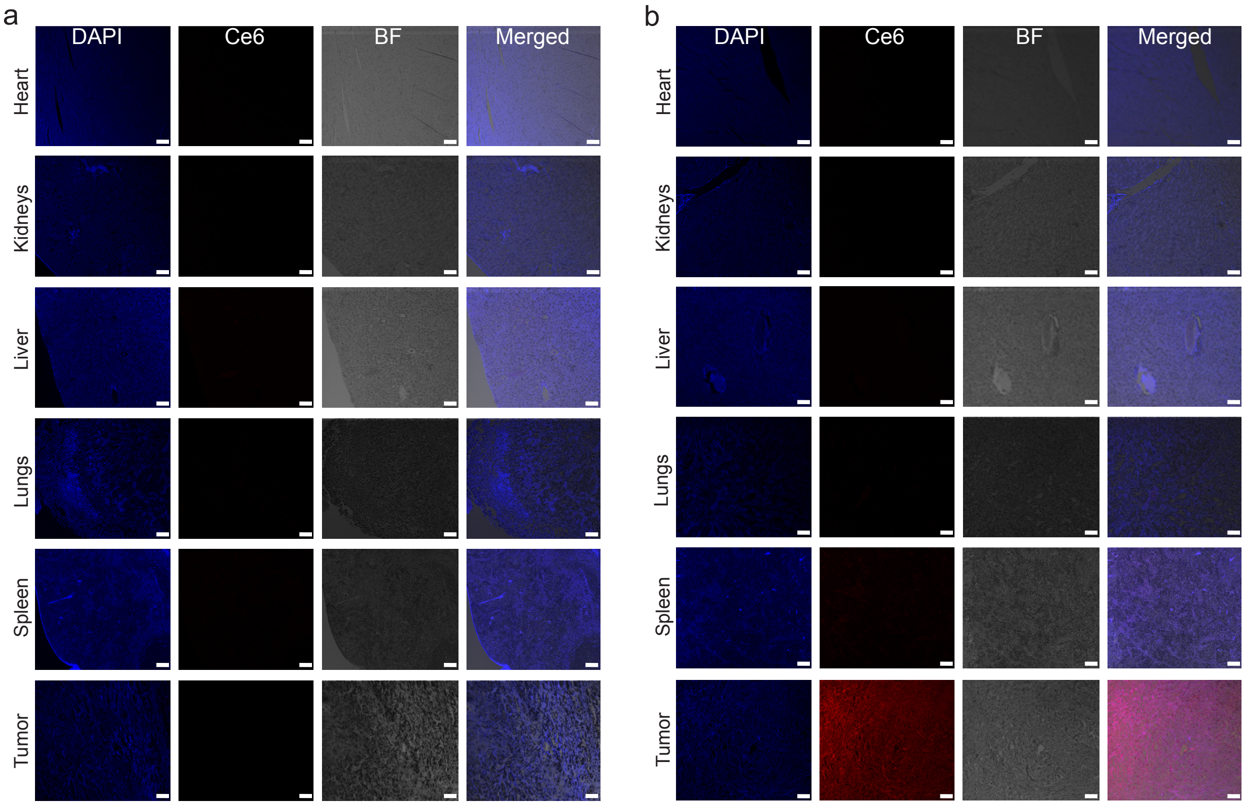

Supporting Figure 14. Fluorescence-based assessment of tumor localization of Ce6-loaded ALUMSNs. Confocal laser scanning microscopy imaging of Ce6 fluorescence in tumor and vital organ sections 8 h after a single *i.v.* injection of saline (**a**) or Ce6-ALUMSNs (11 mg/kg nanospheres, 2.5 mg/kg Ce6) (**b**). Images shown are representative of tissue sections from 4 mice per treatment group. Scale bar = 200 μm.

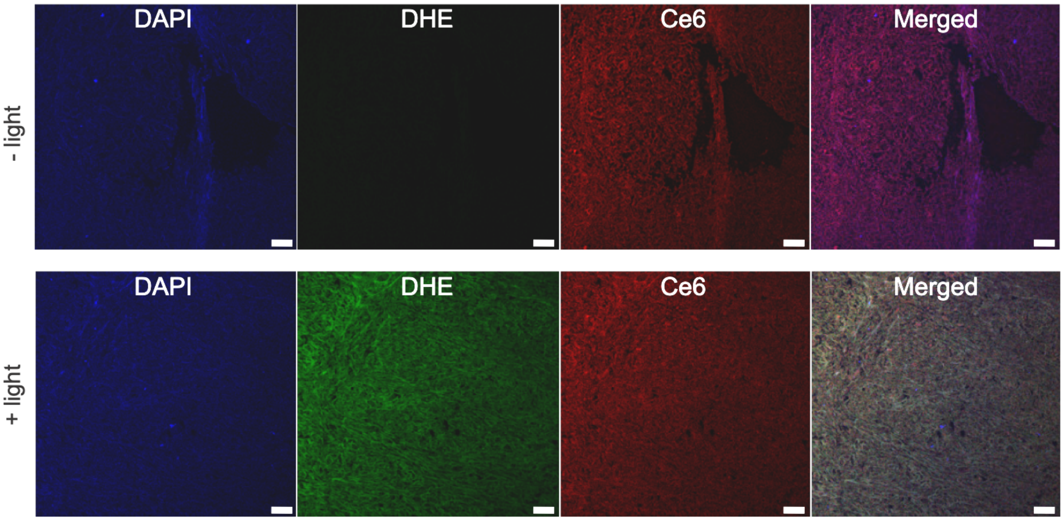

Supporting Figure 15. Detection of reactive oxygen species (ROS) generation in tumors following treatment with Ce6-loaded ALUMSNs and NIR laser irradiation. 4T1 tumor-bearing mice were given a single *i.v.* injection of Ce6-ALUMSNs (11 mg/kg nanospheres, 2.5 mg/kg Ce6). At 8 h post injection, the tumors were stained with the fluorescent ROS probe dihydroethidium (DHE),[10] with (+ light) or without (- light) subsequent exposure to NIR laser irradiation (1.5 W/cm^2, 5 min). The mice were then sacrificed and the tumors were excised, sectioned and imaged using confocal laser scanning microscopy. Images shown are representative of tumor sections from 4 mice per treatment group. Scale bar = 200 µm.

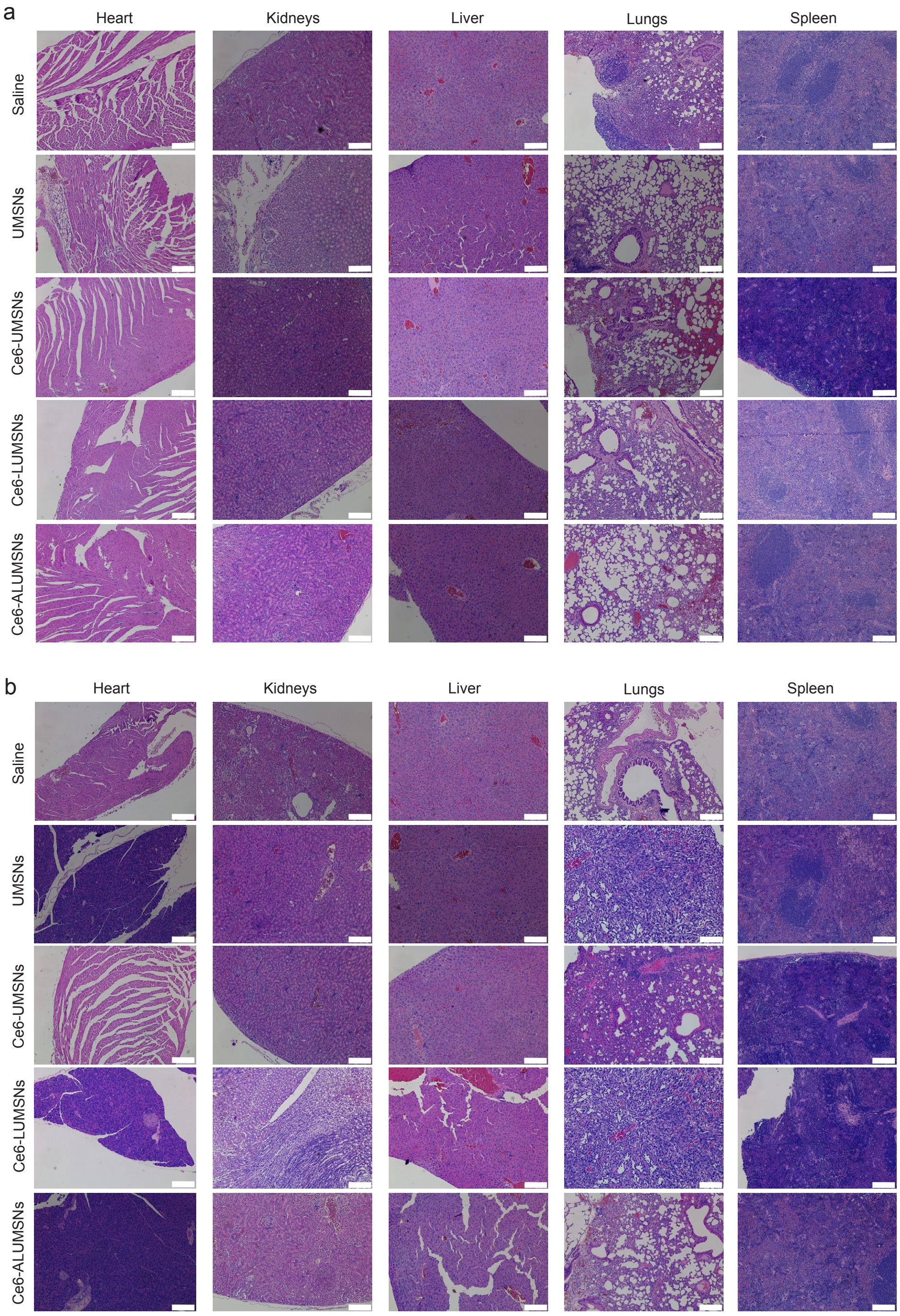

Supporting Figure 16. Histological analysis of vital organs following treatment with the nanospheres. Hematoxylin and eosin (H&E) staining of heart, kidney, liver, lung, and spleen sections from 4T1 tumor-bearing mice after 30 days of treatment (*i.v.* injections administered every 2 days for a total of 15 doses) with saline, UMSNs (11 mg/kg) or Ce6-loaded UMSNs, LUMSNs or ALUMSNs (11 mg/kg nanospheres, 2.5 mg/kg Ce6), in the absence (**a**) or presence (**b**) of NIR laser irradiation (980 nm, 1.5 W/cm^2, 5 min) at 8 h post injection. Images shown are representative of tissue sections from 4 mice per treatment group. Scale bar = 200 µm.

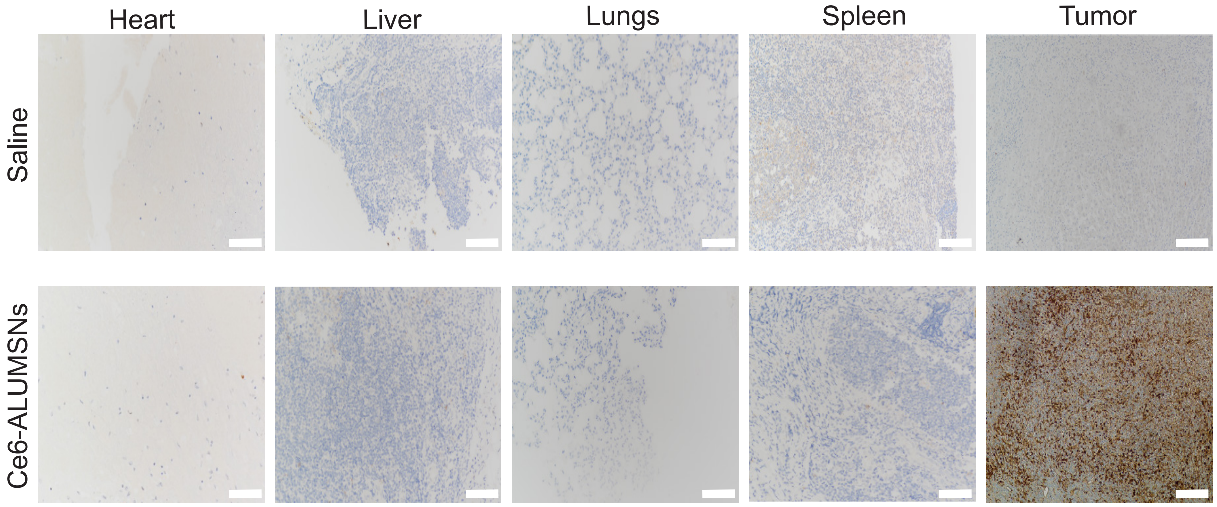

Supporting Figure 17. Immunohistochemistry (IHC) analysis of vital organs following treatment with Ce6-loaded ALUMSNs. Cleaved caspase-3 (a critical mediator of apoptosis)[11] antibody staining of heart, kidney, liver, lung, and spleen sections from 4T1 tumor-bearing mice after 30 days of treatment (*i.v.* injections administered every 2 days for a total of 15 doses) with saline or Ce6-ALUMSNs (11 mg/kg nanospheres, 2.5 mg/kg Ce6) followed by NIR laser irradiation (1.5 W/cm^2, 5 min) at 8 h post injection. Tumor sections are included for comparison. Images shown are representative of tissue sections from 4 mice per treatment group. Scale bar = 200 μm.

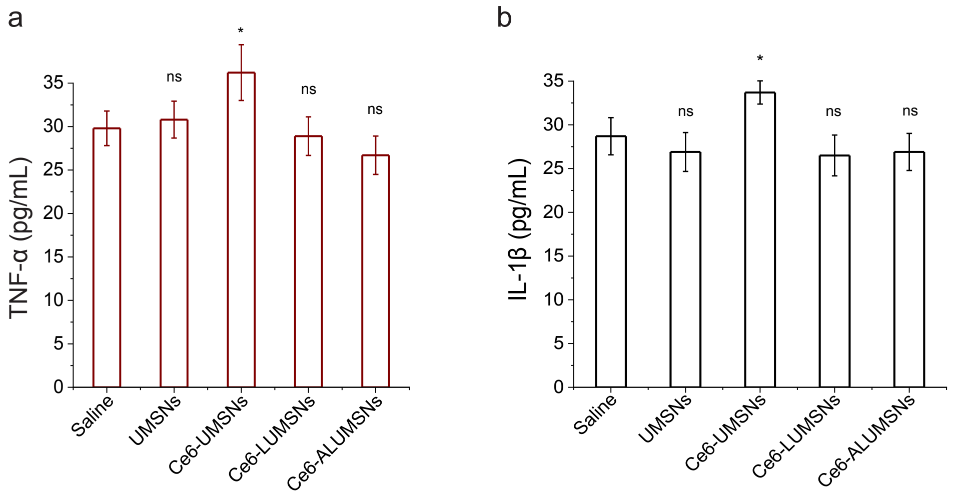

Supporting Figure 18. Quantification of inflammatory cytokines in circulation following treatment with the nanospheres. Measurement of TNF-α (**a**) and IL-1β (**b**) concentrations in serum of test mice following 30 days of treatment (*i.v.* injections administered every 2 days for a total of 15 doses) with saline, UMSNs (11 mg/kg) or Ce6-loaded UMSNs, LUMSNs or ALUMSNs (11 mg/kg nanospheres, 2.5 mg/kg Ce6). TNF-α and IL-1β levels were assayed using commercial ELISA kits ($n = 4$ per group). *$P < 0.05$ or non-significant (ns, $P > 0.05$) for comparisons with the saline-treated controls.

Table 1. Summary of hydrodynamic diameters and zeta potentials of UMSNs, LUMSNs and ALUMSNs.

Nanosphere	Diameter (nm)	Zeta Potential
UMSNs	160 ± 10	-6
LUMSNs	180 ± 10	-20
ALUMSNs	181 ± 10	-11 (pH 7.4)<br>+11 (pH 6.5)

Table 2. Chlorin e6 (Ce6) loading capacity of UMSNs.

Ce6 feed ratio (to 5 mg UMSNs)	Loading capacity (wt%)
0.3	5.0
0.5	10.0
1.0	15.0
1.5	19.0
2.0	22.0

The Ce6 loading capacity of UMSNs was determined as described in the Supporting Experimental Section.

Table 3. Proteins corresponding to the UniProt Knowledgebase (UniProtKB) accession numbers shown in Supporting Figure 5.

UniProtKB Accession Number	Protein
P09486	SPRC
P04732	Metallothionein-1E
Q07869	Peroxisome proliferator-activated receptor alpha
Q03181	Peroxisome proliferator-activated receptor delta
P35527	Keratin, type I cytoskeletal 9
P13645	Keratin, type I cytoskeletal 10
P53634	Dipeptidyl peptidase 1
P04278	Sex hormone-binding globulin
P00338	L-lactate dehydrogenase A chain
Q06828	Fibromodulin
P12259	Coagulation factor V
P99999	Cytochrome c
P07900	Heat shock protein 90 alpha (HSP90α)
P07358	Complement component C8 beta chain
P04406	Glyceraldehyde-3-phosphate dehydrogenase
O60706	ATP-binding cassette, sub-family C member 9 (ABCC9)

Table 4. Serum biochemistry profile of Ce6-loaded ALUMSN-treated test mice.

Analyte[a]	Saline	Ce6-ALUMSNs[b]
AST (U/L)	45.6 ± 0.5	45.0 ± 1.4
ALT (U/L)	45.9 ± 0.4	46.3 ± 1.0
TBIL (mmol/L)	0.4 ± 0.1	0.5 ± 0.1
BUN (mmol/L)	6.8 ± 0.5	6.6 ± 0.3
CRE (mg/dL)	0.4 ± 0.1	0.5 ± 0.1
TRG (mmol/L)	0.5 ± 0.1	0.6 ± 0.1
ALB (g/L)	17.3 ± 0.8	18.2 ± 0.1

[a]Abbreviations: alanine aminotransferase (ALT), aspartate aminotransferase (AST), total bilirubin (TBILI), blood urea nitrogen (BUN), creatinine (CRE), triglycerides (TRG), and albumin (ALB).
[b]ns, non-significant ($P > 0.05$) compared with saline-treated controls ($n = 4$).

SUPPORTING EXPERIMENTAL SECTION

Reagents

Acetone, acetonitrile, ammonium fluoride (NaF, 99.99%), bismuth(III) nitrate pentahydrate ($Bi(NO_3)_3$•$5H_2O$), bovine serum albumin (BSA), calcium chloride, cetyl trimethylammonium bromide (CTAB), chloroform, cyclohexane, dimethyl sulfoxide (DMSO), dithiothreitol (DTT), erbium(III) chloride hexahydrate ($ErCl_3$•$6H_2O$), endocytosis inhibitors (amiloride, chlorpromazine, cytochalasin D and filipin), ethanol, gadolinium(III) chloride hexahydrate ($GdCl_3$•$6H_2O$), hydrochloric acid (HCl), igepal CO-520, indole acetic acid (IAA), mannitol, oleic acid (90%), phorbol 12-myristate 13-acetate (PMA), phosphate-buffered saline (PBS), polyvinylpyrrolidone (PVP), selenium, sodium borohydride ($NaBH_4$), sodium chloride (NaCl), sodium citrate, sodium hydroxide (NaOH), tetraethyl orthosilicate (TEOS), tetramethylrhodamine methyl ester (TMRM), trimethoxy(octadecyl)silane (TMS), Trizma, Trypan Blue, trypsin, Tween 20, ytterbium(III) chloride hexahydrate ($YbCl_3$•$6H_2O$), and yttrium(III) chloride hexahydrate (YCl_3•$6H_2O$) were all purchased from Sigma Aldrich (St. Louis, MO, USA). 2-Aminoethyl dihydrogen phosphate (AEP), ammonia, ammonium bicarbonate buffer, ammonium fluoride, Dead Cell Apoptosis Assay Kit (Alexa 488-conjugated annexin V/propidium iodide (PI)), formic acid, Live/Dead methanol, N-2-hydroxyethylpiperazine-N-2-ethane sulfonic acid (HEPES), octadecane (99%), and Singlet Oxygen Senor Green (SOSG) were purchased from Thermo Fisher (Waltham, MA, USA). Dihydroethidium (DHE) Assay tit was obtained from Abcam (Dallas, TX, USA). 1,2-dipalmitoyl-sn-glycero-3-phosphocholine (DPPC), cholesterol, and the PEGylated derivative of 1,2-distearoyl-sn-glycero-PE (DSPE-PEG_{2000})-maleimide were obtained from Avanti Polar Lipids Inc (Alabaster, AL, USA). Gadolinium-diethylenetriamine pentaacetic acid (Gd-DTPA) was from Schering AG (Berlin, Germany). Cleaved Caspase-3 (Asp175) Antibody (#9661) was acquired from Cell Signaling Technology (Danvers, MA, USA). The CellTiter 96 Aqueous One Solution (MTS) Cell Proliferation Assay Kit was from Promega (Madison, WI, USA). Calcein AM/PI Double Staining Kit, and Human and Mouse Interleukin-1 Beta (Il-1β) and Tumor Necrosis Factor-Alpha (TNF-α) ELISA Kits were obtained from Elabscience (Houston, TX, USA).

Synthesis of the Upconversion Core of the Nanospheres

Initially, the hydrophobic upconversion cores of the nanospheres, which consist of sodium yttrium fluoride doped with lanthanides (ytterbium, erbium, and gadolinium) and bismuth selenide ($NaYF_4$:Yb/Er/Gd,Bi_2Se_3), were synthesized using a thermal decomposition method with oleic acid as a capping agent.[12] YCl_3•$6H_2O$ (0.99 mmol), $YbCl_3$•$6H_2O$ (0.34 mmol), $ErCl_3$•$6H_2O$ (0.06 mmol), and $GdCl_3$•$6H_2O$ (0.61 mmol) were dissolved in an oleic acid (13.4 g)/octadecene (35 mL)

mixture in a round-bottom flask and stirred in an Ar atmosphere. The solution was then evacuated for 45 min until gas production ceased. Subsequently, the reaction mixture was heated to 140 °C in an Ar atmosphere to obtain a yellow-colored solution. The solution was cooled to 45 °C, and sodium hydroxide (150 mg) and ammonium fluoride (300 mg) were added to the reaction mixture and stirred until the solution became clear. The solution was heated to 300 °C in a heating mantle and maintained for 90 min, resulting in discoloration (yellow/brown) of the reaction mixture. 8 mL absolute ethanol was then added to precipitate the upconversion cores, which were separated by centrifugation (4,000×*g*, 25 min) and purified by redispersion and centrifugation in absolute ethanol multiple times. The resulting white powder of upconversion cores was dissolved and stored in cyclohexane.

In order to dope the synthesized upconversion cores with bismuth selenide (Bi_2Se_3), the hydrophobic oleic acid was removed from the surface of the cores using a previously reported acid-induced protocol.[13] Briefly, 5 mL upconversion core solution was diluted in a 15 mL mixture of HCl (2 M) and absolute ethanol (1:1, v/v) and sonicated (40 kHz) for 5 h at 60 °C. The resulting oleic acid-free upconversion cores were collected by centrifugation (4,000×*g*, 25 min), rinsed multiple times with absolute ethanol, and dispersed in Milli-Q water to facilitate subsequent doping with Bi_2Se_3. The selenium precursor was synthesized by reducing selenium (3.16 g, 40 mmol) using $NaBH_4$ (4.54 g, 120 mmol) dissolved in 400 mL Milli-Q water, under an N_2 atmosphere at ambient temperature.[14] Thereafter, Mannitol (1 g) was dissolved in 10 mL Milli-Q water, which was followed by sequential addition of the aqueous solution of oleic acid-free upconversion cores (2 mL, 0.125 mM), ($Bi(NO_3)_3{\bullet}5H_2O$) (0.1 g), and PVP (0.1 g). The mixture was then homogeneously mixed, and the pre-synthesized selenium precursor (0.3 mmol) was added. Following 8 h of continuous stirring, the Bi_2Se_3-doped upconversion cores were collected by centrifugation (4,000×*g*, 25 min), washed with Milli-Q water, and finally dispersed in cyclohexane for further use.

Synthesis of Upconversion Mesoporous Silica Nanospheres (UMSNs)

Enveloping of the upconversion core in a mesoporous silica shell to yield upconversion mesoporous silica nanospheres (UMSNs) was done as previously described.[15] Briefly, 5 mL upconversion core solution (1 mg/mL in cyclohexane) was mixed with 1 mL igepal CO-520, 1 mL ammonia (28 wt%) and 50 mL cyclohexane in a round-bottom flask, and the mixture was sonicated for ~30 min until a transparent emulsion was formed. Following this, 200 µL TEOS and 80 µL TMS were added, and the solution was continuously stirred for 48 h at room temperature. 10 mL acetone was then added to precipitate the UMSNs, which were separated by centrifugation (4,000×*g*, 25 min), washed thrice with absolute ethanol and dried at 60 °C for 12 h. Next, the

synthesized UMSNs were refluxed in a mixture of 10 mL HCl (2 M) and 160 mL methanol for 12 h to remove the surfactants, washed several times with Milli-Q water and freeze dried.[16]

Ce6-loaded UMSNs (Ce6-UMSNs) were produced using a published method for loading mesoporous silica with drugs.[17] UMSNs (5 mg) were dispersed in 0.8 mL Milli-Q water and sonicated until a uniform colloidal solution was obtained. Ce6 (1.5–10 mg) in 0.2 mL DMSO (Supporting Information Table 1) was added to this solution (1 mL total volume) and stirred continuously for 48 h at ambient temperature. The dispersion was then centrifuged (8,000×*g,*10 min) to separate the Ce6-UMSNs (pellet) from free Ce6 (supernatant). The Ce6-UMSNs were vacuum dried, washed thrice with milli-Q water to remove any residual DMSO, and lyophilized for further use. The total mass of Ce6 that was successfully loaded into the UMSNs was calculated by subtracting the mass of residual Ce6 in the isolated supernatant from the initial mass of the photosensitizer used in the nanosphere loading step. The aforementioned masses were determined by UV-Vis spectroscopy (Agilent Cary 4000 UV-Vis Spectrophotometer),[18] using a standard Ce6 concentration calibration curve. The Ce6 loading capacity of UMSNs was determined as follows: loading capacity (%) = $((M_0 - M_s)/W_0) \times 100$, where M_0 and M_s represent the initial mass of Ce6 and mass of Ce6 in the supernatant, respectively, and W_0 represents the mass of Ce6-UMSNs.

Synthesis of Lipid/PEG-Coated UMSNs (LUMSNs)

To coat the nanospheres with a bilayer, first a lipid/PEG film, composed of DPPC/cholesterol/DSPE-PEG_{2000} (at a molar ratio of 77.5:20:2.5),[17] was prepared using an established protocol.[19] Briefly, the components were dissolved in chloroform/ethanol to ensure complete mixing. Thereafter, the solvent was evaporated under a N_2 stream, and the remaining traces of chloroform/ethanol were removed by placing the sample under vacuum for 3 h, resulting in the lipid/PEG film. 10 mg dried UMSNs (Ce6-free or Ce6-loaded) were suspended in 3 mL saline (0.9% NaCl) and sonicated (40 kHz) for 30 s. The suspension was then immediately deposited on the lipid/PEG film at a (Ce6-)UMSN:lipid ratio of 1:1.2 (w/w). Following this, the UMSN-lipid/PEG mixture was probe sonicated (15/15 s on/off cycle, 30 W output power) for 20 min. Finally, the lipid/PEG-coated UMSNs (LUMSNs) were separated from free lipids by centrifugation (10,000×*g*, 10 min), followed by washing twice with saline and Milli-Q water.

Synthesis of ATRAM-Functionalized LUMSNs (ALUMSNs)

In order to facilitate tumor targeting, LUMSNs were functionalized with the targeting acidity-triggered rational membrane (ATRAM) peptide. ATRAM was synthesized using standard Fmoc solid-state protocols by Selleck Chemicals (Houston, TX, USA). The peptide was purified in-house by reversed-phase high-performance liquid chromatography (Waters 2535 QGM HPLC). Subsequently, the peptide's purity was confirmed using mass spectrometry (Agilent 6538 QToF

LC/MS). To synthesize the ATRAM-functionalized LUMSNs (ALUMSNs), we first covalently conjugated DSPE-PEG_{2000}-maleimide with the cysteine residue at the N-terminus of ATRAM.[20] Briefly, ATRAM (600 nmol) was mixed with DSPE-PEG_{2000}-maleimide (500 nmol) in 200 μL methanol, and the mixture was stirred overnight under an N_2 atmosphere at room temperature. Conjugation was confirmed by mass spectrometry. Subsequently, UMSNs (Ce6-free or Ce6-loaded) were coated with DPPC/cholesterol/DSPE-PEG_{2000}-ATRAM (at a molar ratio of 77.5:20:2.5) using the procedure described above.

Characterization of the Nanospheres

The nanospheres were imaged using transmission electron microscopy (TEM) and scanning transmission electron microscopy (STEM) on a Talos F200X (Thermo Fisher). To minimize damage to the nanospheres during imaging (in both TEM and STEM modes), the beam energy was regulated. TEM images were acquired using a 200 kV beam with spot size 5, gun lens 6 and a dose of 1.13–1.16 A/m^2. STEM imaging was done in HAADF (high-angle annular dark-field) mode with the following settings: spot size 9, gun lens 4, and a screen current < 0.2 nA. The composition of the nanospheres was confirmed using STEM-energy dispersive X-ray spectroscopy (STEM-EDS) mapping with the following settings: spot size 6, gun lens 4, and an exposure time of 12.5s. To prepare the samples for TEM/STEM imaging, a drop of nanosphere suspension was placed on a freshly plasma-cleaned 300 mesh copper grid (Ted Pella; Redding, CA, USA) and dried for 2 h at room temperature. The surface area and pore size of the mesoporous silica shell of the nanospheres were analyzed using a 3Flux Adsorption Analyzer (Micrometrics Instruments; Norcross, GA, USA) operated at -196 °C. Size and zeta potential measurements of the nanospheres in different solutions (water; 10 mM phosphate buffer, pH 7.4; 50 mM sodium acetate buffer, pH 5.5; and RPMI 1640 cell culture medium containing 10% FBS, pH 7.4) were carried out on a Zetasizer Nano ZS (Malvern Panalytical; Malvern, UK).

T_1 Relaxation Measurements

T_1 relaxation quantification was performed using a Magnetom Prisma 3T MRI scanner (Siemens Healthineers; Erlangen, Germany) for commercial Gadolinium-diethylenetriamine pentaacetic acid (Gd-DTPA) and UMSNs at various concentrations of Gd (0.0215–0.9526 mM) in 10 mM phosphate buffer (pH 7.4). A saturation-recovery spin echo pulse sequence was employed with a range of 10 repetition times (TR) from 150 ms to 10 s, utilizing the following scanning parameters: echo time (TE), 11 ms; number of averages, 2; field-of-view, 180 mm × 180 mm; acquisition matrix, 320 × 320; in-plane resolution, 0.56 mm × 0.56 mm; and number of slices, 20 slices (slice thickness, 3.5 mm). The T_1 values (measured in milliseconds) were quantified on a pixel-by-pixel basis from Gd-DTPA and UMSNs, at the same Gd concentration, using an in-

house non-linear least-squared fitting script based on the MR relaxation equation implemented in Matlab 2022. The relaxation rate was subsequently calculated as the reciprocal of T_1.[21]

Photodynamic and Photothermal Response

Reactive oxygen species (ROS) generation by Ce6 and Ce6-LUMSNs, at the same PS concentration (0.5 µg/mL, 10 mM phosphate buffer, pH 7.4), following near-infrared (NIR, 980 nm) laser (MDL-H-980-5W diode laser; CNI Optoelectronics, Changchun, China) irradiation (1.0 W/cm^2, 5 min), was monitored using the fluorescent ROS probe Singlet Oxygen Sensor Green (SOSG, 1 µM).[22] SOSG fluorescence ($\lambda_{ex/em}$ = 490/520 nm) was measured on a PerkinElmer LS-55 Fluorescence Spectrometer.

The photothermal response of Ce6-LUMSNs was monitored by measuring the temperature increases following NIR laser irradiation of the nanosphere samples. Ce6-LUMSNs (10–200 µg/mL, 10 mM phosphate buffer, pH 7.4), were irradiated by NIR laser (0.2–1.5 W/cm^2) for 5 min. For comparisons of Ce6-LUMSNs and Ce6, the PS and nanospheres (both at 33 µg/mL Ce6) were subjected to NIR laser irradiation (1.0–1.5 W/cm^2) for 5–10 min. To investigate the photostability of the nanospheres, temperature changes were monitored for 150 µg/mL Ce6-LUMSNs over 5 consecutive NIR laser irradiation (980 nm, 1.5 W/cm^2, 5 min) on/off cycles.[23] Temperatures and thermal images of the samples were recorded on a PI-640i infrared camera (Optris GmBH; Berlin, Germany).

NIR Light-Triggered Cargo Release

Release of the loaded Ce6 from UMSNs and LUMSNs with or without NIR laser irradiation was quantified using a previously published method.[24] Briefly, 50 µg/mL Ce6-UMSNs and Ce6-LUMSNs were loaded into a dialysis bag (Slide-A-Lyzer MINI Dialysis Device, 2000K MWCO, 0.5 mL; Thermo Fisher), which was submerged in 15 mL release medium (10 mM phosphate buffer containing 0.1 wt% Tween 20, pH 7.4) stirring at 75 rpm. The samples were incubated for 24 h, either without or with exposure to NIR laser light of varying irradiation power densities (0.5–1.5 W/cm^2) and durations (1–10 min) at the designated time points (3–16 h) of the experiment. At the indicated times, 0.5 mL of the release medium was removed for analysis, and replenished with the same volume of fresh buffer. The collected samples were centrifuged (1,000×*g*, 5 min) and the resulting pellet of released Ce6 was subsequently dissolved in acetonitrile and assayed by HPLC (Waters 2535 QGM HPLC).[25]

Quantitative Proteomics

To investigate serum protein adsorption to UMSNs and LUMSNs, 1 mg/mL of the nanospheres were incubated in complete cell culture medium (RPMI 1640 containing 10% FBS,

pH 7.4) for 72 h. Thereafter, the adsorbed serum proteins were isolated through centrifugation as previously described.[26,27] The isolated serum proteins were reduced (using 10 mM DTT for 30 min at 85 °C) and alkylated (with 25 mM IAA for 1 h in the dark at room temperature). The protease-specific pH was achieved by diluting the sample with 50 mM ammonium bicarbonate buffer, using a spin filter with a 30-kDa cutoff. The samples were then digested using a MS-grade trypsin/Lys-C protease mix (1:50, w/w) for 24 h at 37 °C. The reaction was quenched (with 1 μL formic acid), and peptide digests were enriched (using offline reversed-phase liquid chromatography (RPLC)), dried, and reconstituted (in a 20 μL solution of 2% acetonitrile/0.1% formic acid) prior to online reversed-phase liquid chromatography-tandem mass spectrometry (RPLC-MS/MS).

RPLC-MS/MS analysis was performed as previously reported.[28] The RPLC was carried out on an UltiMate 3000 RSLCnano System (Dionex) fitted with a C18 column (inner diameter = 75 μm, length = 50 cm; PepMap RSLC). The mobile phases were comprised of 0.1% formic acid (solvent A) and 80% acetonitrile/0.08% formic acid (solvent B). The samples were loaded in solvent A and eluted as follows: 10% B for 5 min, followed by a gradient to 55% B over 40 min and subsequently to 85% B over 5 min, then 85% B for 6 min, and finally re-equilibration with 2% B for 14 min. The LC system was coupled to a Bruker QTOF Impact II mass spectrometer (equipped with an Easy Spray ion source operated in positive ion mode). Full scans were acquired on a TOF MS mass analyzer (*m/z* 200–2200; spectral rate, 2.0 Hz), using the following settings were used: spray voltage, 1.5 kV; dry gas, 3.0 L/min; dry temperature, 165 °C. The auto MS/MS analyses were performed using collision induced dissociation (CID) with a fixed precursor cycle time of 3s. The precursor was released after 0.3 min. The raw files were converted to MGF format by the Bruker Daltonik data analysis software and were searched against reported proteomes with the ProteinScape software using an in-house Mascot search engine (Matrix Science Inc.; Boston, MA). The following search parameters were used: peptide tolerance, 20 ppm; MS/MS tolerance, 0.5 Da; enzyme, trypsin; 2 missed cleavage allowed; and fixed carbamidomethyl modifications of cysteine. Oxidation of methionine and protein N-terminal acetylation were employed as variable modifications. Label-free protein quantification was performed with the MaxQuant software (version 1.6.5.0) using default parameters.[2] The Andromeda search engine was used for searching the raw data against the Universal Protein Resource (UniProt) database.[3,29]

Mitochondrial Membrane Potential ($\Delta\Psi_m$) Measurements

4T1 cells were seeded at a density of 2×10^5 cells/well in 500 μL medium in 4-chambered 35 mm glass bottom Cellview cell culture dishes. After culturing for 24 h at 37 °C in 5% CO_2, the cells were treated with Ce6-ALUMSNs (0.5 μg/mL Ce6) for 4 h at pH 6.5, with or without subsequent exposure to NIR laser irradiation (980 nm, 1.0 W/cm^2, 5 min) following replacement of the medium to remove extracellular nanospheres. The medium was then replaced with fresh

medium containing 200 nM of the $\Delta\Psi_m$ probe TMRM, and the cells were incubated for a further 30 min. Finally, the cells were imaged (Olympus Fluoview FV-1000 confocal laser scanning microscope) and the images were processed using the Fiji software.

Macrophage Recognition, Toxicity and Immunogenicity

Differentiated THP-1 cells were seeded at a density of 1×10^6 cells/well in 6-well plates, cultured for 24 h at 37 °C in 5% CO_2, and subsequently treated with Ce6-loaded UMSNs and ALUMSNs (0.5 µg/mL Ce6) for 4 h at pH 7.4. Thereafter, the cells were washed twice with ice cold PBS, harvested by trypsinization, centrifuged (1,000×*g*, 5 min) and re-suspended in PBS, and uptake of the Ce6-loaded nanospheres was quantified using flow cytometry.

For cell viability studies, THP-1 cells were cultured in standard 96-well plates (5×10^3 cells/well) for 24 h at pH 7.4. The medium was then replaced with fresh medium containing Ce6-UMSNs or Ce6-ALUMSNs (0.05–2 µg/mL Ce6) and the cells were incubated for a further 48 h. Cell viability was assessed using the MTS assay, with the % viability determined from the ratio of the absorbance of the treated cells to the control cells.

To quantify release of tumor necrosis factor-alpha (TNF-α) and interlukin-1 beta (IL-1β), which are inflammatory cytokines primarily produced by macrophages/monocytes during acute inflammation,[30] differentiated THP-1 cells were cultured (2×10^4 cells/well in 100 µL medium in standard 96-well plates) for 24 h at pH 7.4. The medium was then replaced with fresh medium containing Ce6-UMSNs and Ce6-ALUMSNs (0.5 µg/mL Ce6) and the cells were incubated for a further 24 h. The cell culture medium was assayed for secretion of TNF-α and IL-1β using commercial ELISA kits, with cells treated with the macrophage activator lipopolysaccharide (LPS)[8] serving as a positive control, while untreated cells used as a negative control. Total TNF-α and IL-1β levels were determined from the absorbance ($\lambda = 450$ nm) measured on a Synergy H1MF Multi-Mode Microplate-Reader (BioTek; Winooski, VT, USA) using a standard TNF-α concentration calibration curve.

REFERENCES

(1) Antoniak, M. A.; Bazylińska, U.; Wawrzyńczyk, D.; Ćwierzona, M.; Maćkowski, S.; Piątkowski, D.; Kulbacka, J.; Nyk, M. Enhancing Optical Functionality by Co-Loading NaYF4:Yb,Er and CdSe QDs in a Single Core-Shell Nanocapsule. *J. Mater. Chem. C* **2020**, *8* (42), 14796–14804. https://doi.org/10.1039/D0TC03538F.

(2) Tyanova, S.; Temu, T.; Cox, J. The MaxQuant Computational Platform for Mass Spectrometry-Based Shotgun Proteomics. *Nat. Protoc.* **2016**, *11* (12), 2301–2319. https://doi.org/10.1038/nprot.2016.136.

(3) UniProt Consortium. The Universal Protein Resource (UniProt). *Nucleic Acids Res.* **2008**, *36* (Database issue), D190-195. https://doi.org/10.1093/nar/gkm895.

(4) Barltrop, J. A.; Owen, T. C.; Cory, A. H.; Cory, J. G. 5-(3-Carboxymethoxyphenyl)-2-(4,5-Dimethylthiazolyl)-3-(4-Sulfophenyl)Tetrazolium, Inner Salt (MTS) and Related Analogs of 3-(4,5-Dimethylthiazolyl)-2,5-Diphenyltetrazolium Bromide (MTT) Reducing to Purple Water-Soluble Formazans As Cell-Viability Indicators. *Bioorg. Med. Chem. Lett.* **1991**, *1* (11), 611–614. https://doi.org/10.1016/S0960-894X(01)81162-8.

(5) Berridge, M. V.; Tan, A. S. Characterization of the Cellular Reduction of 3-(4,5-Dimethylthiazol-2-Yl)-2,5-Diphenyltetrazolium Bromide (MTT): Subcellular Localization, Substrate Dependence, and Involvement of Mitochondrial Electron Transport in MTT Reduction. *Arch. Biochem. Biophys.* **1993**, *303* (2), 474–482. https://doi.org/10.1006/abbi.1993.1311.

(6) Scaduto, R. C.; Grotyohann, L. W. Measurement of Mitochondrial Membrane Potential Using Fluorescent Rhodamine Derivatives. *Biophys. J.* **1999**, *76* (1 Pt 1), 469–477.

(7) Chanput, W.; Mes, J. J.; Wichers, H. J. THP-1 Cell Line: An in Vitro Cell Model for Immune Modulation Approach. *Int. Immunopharmacol.* **2014**, *23* (1), 37–45. https://doi.org/10.1016/j.intimp.2014.08.002.

(8) Meng, F.; Lowell, C. A. Lipopolysaccharide (LPS)-Induced Macrophage Activation and Signal Transduction in the Absence of Src-Family Kinases Hck, Fgr, and Lyn. *J. Exp. Med.* **1997**, *185* (9), 1661–1670. https://doi.org/10.1084/jem.185.9.1661.

(9) He, Q.; Zhang, Z.; Gao, F.; Li, Y.; Shi, J. In Vivo Biodistribution and Urinary Excretion of Mesoporous Silica Nanoparticles: Effects of Particle Size and PEGylation. *Small* **2011**, *7* (2), 271–280. https://doi.org/10.1002/smll.201001459.

(10) Yang, Z. L.; Tian, W.; Wang, Q.; Zhao, Y.; Zhang, Y. L.; Tian, Y.; Tang, Y. X.; Wang, S. J.; Liu, Y.; Ni, Q. Q.; Lu, G. M.; Teng, Z. G.; Zhang, L. J. Oxygen-Evolving Mesoporous Organosilica Coated Prussian Blue Nanoplatform for Highly Efficient Photodynamic Therapy of Tumors. *Adv. Sci.* **2018**, *5* (5), 1700847. https://doi.org/10.1002/advs.201700847.

(11) Chen, D. L.; Engle, J. T.; Griffin, E. A.; Miller, J. P.; Chu, W.; Zhou, D.; Mach, R. H. Imaging Caspase-3 Activation as a Marker of Apoptosis-Targeted Treatment Response in Cancer. *Mol. Imaging Biol. MIB Off. Publ. Acad. Mol. Imaging* **2015**, *17* (3), 384–393. https://doi.org/10.1007/s11307-014-0802-8.

(12) Klier, D. T.; Kumke, M. U. Upconversion NaYF4:Yb:Er Nanoparticles Co-Doped with Gd3+ and Nd3+ for Thermometry on the Nanoscale. *RSC Adv.* **2015**, *5* (82), 67149–67156. https://doi.org/10.1039/C5RA11502G.

(13) Zhao, S.; Tian, R.; Shao, B.; Feng, Y.; Yuan, S.; Dong, L.; Zhang, L.; Wang, Z.; You, H. UCNP–Bi2Se3 Upconverting Nanohybrid for Upconversion Luminescence and CT Imaging

and Photothermal Therapy. *Chem. – Eur. J.* **2020**, *26* (5), 1127–1135. https://doi.org/10.1002/chem.201904586.

(14) Han, C.; Sun, Q.; Cheng, Z. X.; Wang, J. L.; Li, Z.; Lu, G. Q. (Max); Dou, S. X. Ambient Scalable Synthesis of Surfactant-Free Thermoelectric CuAgSe Nanoparticles with Reversible Metallic- *n-p* Conductivity Transition. *J. Am. Chem. Soc.* **2014**, *136* (50), 17626–17633. https://doi.org/10.1021/ja510433j.

(15) Xu, F.; Ding, L.; Tao, W.; Yang, X.; Qian, H.; Yao, R. Mesoporous-Silica-Coated Upconversion Nanoparticles Loaded with Vitamin B12 for near-Infrared-Light Mediated Photodynamic Therapy. *Mater. Lett.* **2016**, *167*, 205–208. https://doi.org/10.1016/j.matlet.2015.12.105.

(16) Palanikumar, L.; Choi, E. S.; Cheon, J. Y.; Joo, S. H.; Ryu, J.-H. Noncovalent Polymer-Gatekeeper in Mesoporous Silica Nanoparticles as a Targeted Drug Delivery Platform. *Adv. Funct. Mater.* **2015**, *25* (6), 957–965. https://doi.org/10.1002/adfm.201402755.

(17) Meng, H.; Wang, M.; Liu, H.; Liu, X.; Situ, A.; Wu, B.; Ji, Z.; Chang, C. H.; Nel, A. E. Use of a Lipid-Coated Mesoporous Silica Nanoparticle Platform for Synergistic Gemcitabine and Paclitaxel Delivery to Human Pancreatic Cancer in Mice. *ACS Nano* **2015**, *9* (4), 3540–3557. https://doi.org/10.1021/acsnano.5b00510.

(18) Liu, H.; Laan, A. C.; Plomp, J.; Parnell, S. R.; Men, Y.; Dalgliesh, R. M.; Eelkema, R.; Denkova, A. G. Ionizing Radiation-Induced Release from Poly(ε-Caprolactone-b-Ethylene Glycol) Micelles. *ACS Appl. Polym. Mater.* **2021**, *3* (2), 968–975. https://doi.org/10.1021/acsapm.0c01258.

(19) Mukundan, V.; Maksoudian, C.; Vogel, M. C.; Chehade, I.; Katsiotis, M. S.; Alhassan, S. M.; Magzoub, M. Cytotoxicity of Prion Protein-Derived Cell-Penetrating Peptides Is Modulated by PH but Independent of Amyloid Formation. *Arch. Biochem. Biophys.* **2017**, *613*, 31–42. https://doi.org/10.1016/j.abb.2016.11.001.

(20) Yao, L.; Daniels, J.; Wijesinghe, D.; Andreev, O. A.; Reshetnyak, Y. K. PHLIP®-Mediated Delivery of PEGylated Liposomes to Cancer Cells. *J. Controlled Release* **2013**, *167* (3), 228–237. https://doi.org/10.1016/j.jconrel.2013.01.037.

(21) Nicholls, F. J.; Rotz, M. W.; Ghuman, H.; MacRenaris, K. W.; Meade, T. J.; Modo, M. DNA-Gadolinium-Gold Nanoparticles for in Vivo T1 MR Imaging of Transplanted Human Neural Stem Cells. *Biomaterials* **2016**, *77*, 291–306. https://doi.org/10.1016/j.biomaterials.2015.11.021.

(22) Flors, C.; Fryer, M. J.; Waring, J.; Reeder, B.; Bechtold, U.; Mullineaux, P. M.; Nonell, S.; Wilson, M. T.; Baker, N. R. Imaging the Production of Singlet Oxygen in Vivo Using a New Fluorescent Sensor, Singlet Oxygen Sensor Green. *J. Exp. Bot.* **2006**, *57* (8), 1725–1734. https://doi.org/10.1093/jxb/erj181.

(23) Ma, N.; Zhang, M.-K.; Wang, X.-S.; Zhang, L.; Feng, J.; Zhang, X.-Z. NIR Light-Triggered Degradable MoTe2 Nanosheets for Combined Photothermal and Chemotherapy of Cancer. *Adv. Funct. Mater.* **2018**, *28* (31), 1801139. https://doi.org/10.1002/adfm.201801139.

(24) Yang, J.; Teng, Y.; Fu, Y.; Zhang, C. Chlorins E6 Loaded Silica Nanoparticles Coated with Gastric Cancer Cell Membrane for Tumor Specific Photodynamic Therapy of Gastric Cancer. *Int. J. Nanomedicine* **2019**, *Volume 14*, 5061–5071. https://doi.org/10.2147/IJN.S202910.

(25) Isakau, H. A.; Trukhacheva, T. V.; Zhebentyaev, A. I.; Petrov, P. T. HPLC Study of Chlorin E6 and Its Molecular Complex with Polyvinylpyrrolidone. *Biomed. Chromatogr.* **2007**, *21* (3), 318–325. https://doi.org/10.1002/bmc.762.

(26) Shevchenko, A.; Tomas, H.; Havli, J.; Olsen, J. V.; Mann, M. In-Gel Digestion for Mass Spectrometric Characterization of Proteins and Proteomes. *Nat. Protoc.* **2006**, *1* (6), 2856–2860. https://doi.org/10.1038/nprot.2006.468.

(27) Oh, J. Y.; Kim, H. S.; Palanikumar, L.; Go, E. M.; Jana, B.; Park, S. A.; Kim, H. Y.; Kim, K.; Seo, J. K.; Kwak, S. K.; Kim, C.; Kang, S.; Ryu, J.-H. Cloaking Nanoparticles with Protein Corona Shield for Targeted Drug Delivery. *Nat. Commun.* **2018**, *9* (1), 4548. https://doi.org/10.1038/s41467-018-06979-4.

(28) Ali, L.; Flowers, S. A.; Jin, C.; Bennet, E. P.; Ekwall, A.-K. H.; Karlsson, N. G. The O-Glycomap of Lubricin, a Novel Mucin Responsible for Joint Lubrication, Identified by Site-Specific Glycopeptide Analysis. *Mol. Cell. Proteomics MCP* **2014**, *13* (12), 3396–3409. https://doi.org/10.1074/mcp.M114.040865.

(29) Cox, J.; Neuhauser, N.; Michalski, A.; Scheltema, R. A.; Olsen, J. V.; Mann, M. Andromeda: A Peptide Search Engine Integrated into the MaxQuant Environment. *J. Proteome Res.* **2011**, *10* (4), 1794–1805. https://doi.org/10.1021/pr101065j.

(30) Tsarouchas, T. M.; Wehner, D.; Cavone, L.; Munir, T.; Keatinge, M.; Lambertus, M.; Underhill, A.; Barrett, T.; Kassapis, E.; Ogryzko, N.; Feng, Y.; Ham, T. J. van; Becker, T.; Becker, C. G. Dynamic Control of Proinflammatory Cytokines Il-1β and Tnf-α by Macrophages in Zebrafish Spinal Cord Regeneration. *Nat. Commun.* **2018**, *9* (1), 1–17. https://doi.org/10.1038/s41467-018-07036-w.
